# Supplementary material for: Internet-based support for informal caregivers to individuals with head and neck cancer (Carer eSupport): a study protocol for the development and feasibility testing of a complex online intervention
Source: BMJ Open. 2022 May 27;12(5):e057442. doi: 10.1136/bmjopen-2021-057442 (PMC9150150; doi:10.1136/bmjopen-2021-057442)
Supplement: Author's manuscript [file bmjopen-2021-057442.draft_revisions.pdf]

BMJ Open is committed to open peer review. As part of this commitment we make the peer review history of every article we publish publicly available.

When an article is published we post the peer reviewers' comments and the authors' responses online. We also post the versions of the paper that were used during peer review. These are the versions that the peer review comments apply to.

The versions of the paper that follow are the versions that were submitted during the peer review process. They are not the versions of record or the final published versions. They should not be cited or distributed as the published version of this manuscript.

BMJ Open is an open access journal and the full, final, typeset and author-corrected version of record of the manuscript is available on our site with no access controls, subscription charges or pay-per-view fees (http://bmjopen.bmj.com).

If you have any questions on BMJ Open's open peer review process please email info.bmjopen@bmj.com

BMJ Open

Carer-eSupport: Rationale and design of internet-based support for informal caregivers of individuals with head and neck cancer – a study protocol

Journal:	*BMJ Open*
Manuscript ID	bmjopen-2021-057442
Article Type:	Protocol
Date Submitted by the Author:	21-Sep-2021
Complete List of Authors:	Langegård, Ulrica; Uppsala Universitet, Experimental and Clinical Oncology, Dept. of Immunology, Genetics and Pathology Uppsala, SE<br>Cajander, Åsa; Uppsala Universitet Teknisk-naturvetenskapliga fakulteten, Department of Information Technology; Uppsala University<br>Carlsson, Maria; Department of Public Health and Caring Sciences, Caring Sciences<br>von Essen, Louise; Uppsala Universitet, Clinical Psychology in Healthcare, Department of Women's and Children's Health<br>Ahmad, Awais; Uppsala Universitet Teknisk-naturvetenskapliga fakulteten, Department of Information Technology Lägervyddsvägen 2 Uppsala, FIN (+358), SE 75337<br>Laurell, Göran; Department of Surgical Sciences, Otorhinolaryngology and head and neck surgery, Uppsala University Sweden.<br>Tiblom Ehrsson, Ylva; Department of Surgical Sciences, Otorhinolaryngology and head and neck surgery, Uppsala University Sweden<br>Johansson, Birgitta; Uppsala Universitet, Department of Public Health and Caring Sciences, Lifestyle and rehabilitation in long-term illness; Uppsala Universitet, Experimental and Clinical Oncology, Dept. of Immunology, Genetics and Pathology
Keywords:	ONCOLOGY, Protocols & guidelines < HEALTH SERVICES ADMINISTRATION & MANAGEMENT, RADIOTHERAPY, Adult radiotherapy < RADIOTHERAPY, QUALITATIVE RESEARCH, Head & neck tumours < ONCOLOGY

SCHOLARONE™
Manuscripts

Carer-eSupport: Rationale and design of internet-based support for informal caregivers of individuals with head and neck cancer – a study protocol

Ulrica Langegård, PhD, RN[1]; Åsa Cajander, PhD, Professor[2;] Maria Carlsson, PhD, Assoc Professor, RN[3]; Louise von Essen, PhD, Professor, Lic Psychologist[4]; Awais Ahmad, PhD student[2,] Göran Laurell, PhD, Professor, MD[5], Ylva Tiblom Ehrsson, PhD, RN[5*], Birgitta Johansson, PhD, Assoc Professor, RN[1*]

[1]Department of Immunology, Genetics and Patology, *Experimental and Clinical Oncology,* Uppsala University, Sweden.

[2] Department of Information Technology, Division of Visual Information and Interaction, Uppsala University, Sweden.

[3]Department of Public Health and Caring Sciences, Lifestyle and rehabilitation in long term illness, Uppsala University, Sweden.

[4] Department of Women´s and Children´s Health, Healthcare sciences and e-Health, Uppsala University, Sweden.

[5]Department of Surgical Sciences, Otorhinolaryngology and head and neck surgery, Uppsala University Sweden.

*equal contribution

Email addresses:

ulrica.langegard@igp.uu.se

asa.cajander@it.uu.se

maria.carlsson@pubcare.uu.se

louise-von.essen@kbh.uu.se

awais.ahmad@it.uu.se

goran.laurell@surgsci.uu.se

ylva.tiblom.ehrsson@surgsci.uu.se

birgitta.johansson@igp.uu.se

For peer review only

Corresponding author:
Ulrica Langegård, Department of immunology, genetics and patology, Uppsala University
Blod- och tumörsjukdomar, Administration
751 85 Uppsala
Telephone: +46 729-999020
Email: ulrica.langegard@igp.uu.se
ORCID id: orcid.org/0000-0001-8174-579X

Responsible for the trial

Birgitta Johansson, PhD, Assoc Professor, RN
Department of immunology, genetics and patology, Uppsala university
Tel/Phone. +46 70425 09 12
ORCID: 0000-0001-6226-6849

Keywords: study protocol, development, feasibility testing, complex intervention, head and neck cancer, informal caregiver

Word count: 4027

For peer review only

Carer-eSupport: Rationale and design of internet-based support for informal caregivers of individuals with head and neck cancer – a study protocol

Abstract

Introduction

Informal caregivers may play a vital role in the care and support of patients with cancer. The shift from inpatient to outpatient cancer care has resulted in a transition of care from professional to informal caregivers. This has increased the complexity of caregivers' responsibilities, which they may not be prepared for. There is a need to support informal caregivers to prepare them for caregiving, decrease caregiver burden, and prevent declining health. The project aims are to develop a user friendly internet-based intervention (Carer-eSupport) for informal caregivers of individuals with head and neck cancer and test its feasibility and acceptability.

Methods and analysis

This is a multicentre study involving the ear, nose, and throat clinics and the oncology and radiotherapy clinics at three university hospitals. The study protocol comprises two phases, *development* and *feasibility testing*, using the Medical Research Council framework for developing a complex intervention. Carer-eSupport will be based on the results from focus group with informal caregivers and healthcare professionals, scientific evidence, the Social Cognitive Theory and the Theory of Acceptance and Use of Technology. The goal is to develop a feasible and acceptable internet-based intervention to be evaluated in a forthcoming randomized controlled trial regarding effects on self-reported preparedness for caregiving, caregiving burden, and informal caregiver health.

Ethics and dissemination

All procedures have been approved by the Ethics committee at Uppsala University (Dnr: 2020-04650). Informed consent will be obtained before enrolment of patients, their informal caregivers, and healthcare staff. The feasibility testing is registered at Clinicaltrials.gov (Identifier: NCT05028452). Findings will be disseminated in peer-reviewed journal publications.

For peer review only

Article Summary

Strengths and limitations of the study

- This protocol describes a study which, to the best of our knowledge, is the first to develop and test the feasibility of internet-based support for informal caregivers of patients with head and neck cancer.
- Carer-eSupport will be developed alongside informal caregivers and healthcare professionals, based on scientific evidence and established theories.
- The feasibility and usability of Carer-eSupport will be tested using both quantitative and qualitative data.
- A solid targeted review will be performed, though this may result in loss of valuable research data in comparison to a systematic review.
- Head and neck cancer encompasses a heterogeneous group of diagnoses in the upper aerodigestive tract and those affected have differing needs and prognoses, creating a potential challenge in meeting informal caregivers' varying needs and expectations.

For peer review only

Carer-eSupport: Rationale and design of internet-based support for informal caregivers of individuals with head and neck cancer – a study protocol

Introduction

Informal caregivers (ICs) are crucial in the care and support of patients with cancer. An IC may be a relative, friend, or partner who provides (physical/emotional/practical) assistance to a patient[1]. The shift from inpatient to outpatient cancer care has resulted in a transition of care to ICs, increasing the complexity of their responsibilities, which they may not be prepared for. Thus, ICs may need support.

Patients with head and neck cancer and their ICs

Over 800,000 people worldwide were diagnosed with head and neck cancer (HNC) in 2018[2]. HNC comprises several diagnoses with differing prognoses, and about 9.8% of those affected die within six months of diagnosis, due to tumor-, patient-, or treatment-related factors[3]. Many individuals with HNC experience long-term physical and/or psychological distress, such as eating problems, pain, and depressive symptoms[4-7]. Unmet needs have been identified among 60–70% of patients and a similar proportion of their IC [8]. The ICs responsibilities of dysphagia management, including preparation of special meals and tube feeding, are time-consuming and may affect ICs' health negatively [9, 10]. A recent study of ICs to individuals with HNC revealed that the perceived difficulty of caregiving tasks was a more crucial driver of psychological distress than the number of tasks or patient experiences of symptom clusters [11]. A systematic review of the psychological health of ICs to individuals with HNC concluded that ICs suffer from high levels of fear of cancer recurrence,[12] and experience psychological distress and depressive disorders six months after diagnosis [13]. Taken together, this indicates a need to develop support interventions for ICs to individuals with HNC.

A rationale for Carer-eSupport

Preparedness and caregiver burden

Preparedness for caregiving can be defined as ICs' self-perceived capacity to provide physical, emotional, or practical care and manage the stresses of caregiving.[14] Lacking preparedness may increase caregiver burden, which can be described as a multidimensional

For peer review only - http://bmjopen.bmj.com/site/about/guidelines.xhtml

biopsychosocial reaction resulting from an imbalance between care demands and a caregiver's resources to care [15]. The caregiver burden may encompass physical, mental, and financial components and social isolation [16-22]. High perceived preparedness is associated with a lower caregiver burden and may prevent declining IC health [23].

Caregiver interventions

The effectiveness of interventions focusing on the ability to care for patients with cancer has been evaluated in several systematic reviews [24-32]. Northouse et al.[26] found that interventions for ICs increased preparedness for caregiving, reduced caregiver burden and anxiety, and improved family relationships. Applebaum et al.[28] concluded that interventions combining different types of support, e.g., skill-building and psychoeducation, including support to cope with the illness, seemed to offer the greatest benefits. Recently, Ferrell et al. [25] showed that pain management skills are particularly important for caregivers, and suggested a focus on interventions focusing on general physical care and symptom assessment. A combination of interventions including instruction assessing side effects, techniques for assisting in pain management, and general training in problem-solving skills may increase ICs' preparedness for caregiving [25]. Treanor, [32] in a recent review, suggested similar strategies to reduce the burden for ICs of individuals affected by cancer. However, while various skill-based interventions have been evaluated, it remains unknown which approach has the best effects on ICs preparedness for care and care burden.

Internet-based interventions

Internet-based interventions need to be further studied, especially as regards effects on caregivers' preparedness for caregiving, physical health, and social and psychological outcomes [33, 34]. There is a large variation in perceived meaningfulness of internet-based activities, highlighting the importance of adapting interventions to the needs and preferences of the intended end users. Willingness to use internet-based interventions depends on early and thorough involvement of end users in design and development [35-37]. Ugalde et al.[38] discussed the lack of actions to facilitate implementation in clinical settings. Crucial characteristics of a successful intervention are its relevance for the target groups and its potential to be effectively implemented in clinical care, provided that it is effective. Craig et al.[39] highlighted that early and continuous involvement of stakeholders and end users in the development and feasibility testing of an intervention is necessary to achieve these goals. Thus, involvement of end users in the development and testing of Carer-eSupport is e pivotal.

For peer review only - http://bmjopen.bmj.com/site/about/guidelines.xhtml

The increasing use of artificial intelligence (AI) has created new opportunities for personalised and predictive healthcare systems [40, 41]. Carer-eSupport can potentially be integrated with AI to provide ICs with relevant, accurate information, using users' experiences and data to increase ICs' preparedness for caregiving and decrease caregiver burden.

Theoretical framework

The Social Cognitive Theory (SCT) [42] is used as a theoretical framework for the development of Carer-eSupport. The foundation for SCT is the significance of the interplay between behaviours and personal/social/environmental factors [42]. Self-efficacy is a core construct in SCT, conceptualised as a person's self-perceived capacity to perform in a particular situation [43]. In the context of caring for patients with HNC, it can be assumed that ICs with high self-efficacy will perceive themselves as prepared to perform caregiving tasks, and therefore will be successful in doing so. SCT is supplemented with the well-established Unified Theory of Acceptance and Use of Technology (UTAUT) framework [44]. Technology acceptance models have been designed to predict behaviours and estimate acceptance and satisfaction in individuals using technology. UTAUT includes four relevant constructs: performance expectancy, effort expectancy, social influence, and facilitating conditions. In this study, the constructs are given the following meanings:

1. Performance expectancy refers to whether ICs believe that Carer-eSupport can improve their preparedness for care.
2. Effort expectancy refers to the efforts ICs expect when using Carer-eSupport.
3. Social influence refers whether important people in an IC's surroundings believe the IC should use the intervention.
4. Facilitating conditions are perceived enablers and barriers that influence an IC's understanding of the ease or challenges of using Carer-eSupport. These could be existing organizational or technical infrastructures that support use of the system.

In summary, the needs of ICs in giving support to individuals with HNC and factors of importance for their usage of and adherence to internet-based support remain to be explored. Systematic reviews suggest that interventions integrating multiple evidence-based modalities may increase ICs' preparedness for caregiving, decrease their caregiver burden, and improve their health. Interventions must fulfil ICs' needs so they can provide the best possible patient

For peer review only - http://bmjopen.bmj.com/site/about/guidelines.xhtml

care that their resources permit. Thus, internet-based interventions for ICs should be developed, tested, and evaluated in collaboration with ICs and healthcare professionals.

Aims

The aims are to describe the development of a user-friendly internet-based intervention (Carer-eSupport) for improving ICs' ability to support individuals with HNC and to determine the intervention's feasibility and acceptability. An additional aim is to explore healthcare professionals' perceptions of the content and design of Carer-eSupport.

Research questions are:

- Which support is relevant and acceptable to ICs of individuals with HNC?
- How should Carer-eSupport be designed to improve utility and optimize the user experience for ICs?
- How do healthcare professionals perceive the content and design of Carer-eSupport?
- To what extent do ICs use Carer-eSupport?
- What is the relevance, acceptability, utility, and user experience of Carer-eSupport?

Methods and analysis

Design

The study protocol is designed in accordance with the Medical Research Council (MRC) framework, [45] which includes four critical phases: (I) development, (II) feasibility testing, (III) evaluation, and (IV) implementation. The present protocol describes the development and feasibility testing of Carer-eSupport (see Figure 1). This protocol complies with the SPIRIT guidelines on writing protocols (see Additional file 1)[46].

Setting and procedure phases I and II

Ear nose and throat (ENT)-, oncology and radiotherapy clinics at three Swedish university hospitals will be involved. Patients with HNC and their ICs will be identified by the healthcare professionals responsible for their care. ICs are designated by the patients, who must provide informed consent before their ICs can be approached. Healthcare professionals who are asked to participate in the study will receive information about the study from a contact person at the hospital where they work, who will provide contact details to a project researcher. Next, ICs and healthcare professionals will be contacted with more information

For peer review only

about the study by a member of the research team (UL). They will be invited to participate and asked to provide informed consent.

Phase I - Development

Phase I encompasses the development of Carer-eSupport. This will be based on a literature review, a theoretical framework, focus group discussion (FGDs) with ICs and healthcare professionals, development of a first version of Carer eSupport, and interviews regarding healthcare professionals' perception of this first version.

Literature review and theoretical framework

A thorough review of the literature on the complexity of being an IC to a patient with cancer, in particular a patient with HNC, has been conducted and is presented in the introduction above. The literature review findings, SCT, [42] and UTAUT [44] will guide the development of a semi-structured interview guide for FGDs with ICs to patients with HNC.

Focus groups discussions

Sample of ICs

ICs are designated by patients, their eligibility is assessed as follows.

Inclusion criteria

- IC to a person with HNC about to start treatment, under treatment, or who has completed treatment not more than three months earlier.
- Age >18 years.

Exclusion criteria

- Confusion or dementia.
- Unable to understand, speak, and read Swedish.

People of different genders and ages, with experience of being an IC to an individual with HNC, will be invited – to form one or two groups of 6-8 ICs at each of the three study sites.

Sample of healthcare professionals

The healthcare professional groups will include people of different genders, ages, and professions, e.g., nurses, assistant nurses, physicians, psychologists, dietitians, speech therapists, and dental hygienists. At least one group of 6–8 healthcare professionals at each of the three study sites will be included. The inclusion criteria is having experience of caring for patients with HNC.

For peer review only - http://bmjopen.bmj.com/site/about/guidelines.xhtml

The semi-structured interviews

The FGDs with ICs [47, 48] will be divided into two parts. The first will focus on ICs' perceived experiences and needs. This may include knowledge and skills related to caregiving of individuals with HNC and support that could decrease the caregiver burden and increase ICs' preparedness for caregiving and ability to consider their own health. Self-care strategies and the need for psychosocial counselling will be explored. During the FGDs, open questions will be used to address ICs' experiences, e.g., "What is/has been the major challenge for you as an IC to an individual with HNC?", "What kind of support do/did you need as an IC to a person with HNC?", "What support do/did you get that meets/met your needs?", "What support do/did you feel is/was lacking in your role as an IC?".

The second part will explore the ICs' positive and negative experiences of internet-based support and how it should be designed to have high utility and an optimal user experience. It will also explore ICs' thoughts on integration of AI into Carer-eSupport, for example, whether it's content should be automatically adapted depending on how an IC uses it. If necessary, additional individual interviews will be performed.

The FGDs with healthcare professionals will be used to understand how Carer-eSupport should be designed to be useful for ICs from a health care professionals perspective. Healthcare professionals' perceptions of ICs' needs for support concerning caregiving and wellbeing, as well as their views on how such support can be delivered via the internet in a clinical setting, will be explored. The interview guide for healthcare professionals contains questions such as "What support do ICs to individuals treated for HNC need?", "Which professions are needed in the support of the ICs?", and "How do you imagine that an internet-based support for ICs will be useful in clinical practice?"

Researchers (1facilitator and 1 moderator) and a PhD student will conduct the focus groups and notes will be taken by the moderator (UL), to capture participants' non-verbal expressions. The FGDs will be conducted online using end-to-end encryption Zoom software with assurance that no unauthorized person can entrance the meeting. Zoom servers (San Jose, USA) in European Union are used for Swedish Universities and is compliant with General Data Protection Regulations, GDPR [49] according to Swedish University regulations. The FGDs are expected to last 120–140 minutes, and will be audiotaped and transcribed.

For peer review only - http://bmjopen.bmj.com/site/about/guidelines.xhtml

Qualitative analysis of the focus group discussions

The transcribed interviews and notes from these will be analyzed using qualitative content analysis [50]. To ensure the trustworthiness of findings, researchers will use member checking and group debriefing, as described by Lincoln and Guba [51]. To confirm that the analysis is rigorous, the researchers will write detailed notes on the emergent analytical and theoretical insights.

Design and delivery of a first version of Carer-eSupport

Carer-eSupport will be provided via a web portal developed within the Uppsala University Psychosocial Care Program (U - CARE) [52, 53]. The U-CARE-portal (Portal) has been developed and refined since 2010 and has been used in several previous projects. The Portal is designed for building and delivery of interventions and has several additional functions for instance for collecting informed consent online, performing randomization in studies, collecting self-report data, sending reminders at specific times, and logging user activity. The Portal uses a secure login process based on electronic identification and all data are handled in a secure manner. Carer-eSupport will be developed in parallel with the analyses of the FGDs, but most of the development will have been performed before the analyses are completed. The research group has extensive knowledge of development, testing, and evaluation of internet-based support, including usability and user experience, and will have the main responsibility for development.

The healthcare professionals' perceptions of the first version of Carer-eSupport

The healthcare professionals will be given access to the first version of Carer-eSupport via the Portal for one month. Thereafter, individual interviews will be held with them regarding the features of Carer-eSupport, its content and design, and if they encountered any technical problems. The interviews will be conducted online by the researchers, using end-to-end encryption Zoom software (San Jose, USA).

Phase II - Feasibility testing

Phase II encompasses testing the relevance, acceptability, utility, and user experience of Carer-eSupport. [45].

Design

In phase II, Carer-eSupport will be tested in an one arm feasibility study with a posttest design.

For peer review only - http://bmjopen.bmj.com/site/about/guidelines.xhtml

Sample of ICs

The recommendations of Cocks et al. on sample size in feasibility studies [54] would mean that approximately thirty ICs to patients with HNC need to be included to critically review the relevance and acceptability of the content, usability, and usefulness of Carer-eSupport.

Inclusion criteria

- IC to an individual with HNC about to start treatment, under treatment, or who has completed treatment not more than one month earlier.
- Age >18 years.

Exclusion criteria

- Confusion or dementia.
- Unable to understand, speak, and read Swedish.

The test version of Carer-eSupport

ICs will be given access to Carer-eSupport for one month. The support is suggested to be delivered in the form of electronic live education for ICs and a library of written and audiovisual material. It will be possible to send messages and have conversations via video link with healthcare professionals, use discussion forums, and use digital rooms to exchange experiences with other ICs in a similar situation.

Data collection

Feasibility testing

Carer-eSupport will be considered feasible if ≥45% of eligible, approached ICs are recruited in participation, ≥50% of included ICs use the support (based on number of logins, use of educational materials according to log data and individual interviews), attrition is <30%, and ≥50% complete the questionnaires[54].

Individual interviews

Individual semi-structured interviews with ICs will be conducted after the test period. The interviews will include questions on recruitment to the study and experiences of Carer-eSupport (e.g., components, functions, layout, technical problems), in order to optimize its relevance and acceptability. The individual interviews will be conducted online by the researchers, using end-to-end encryption Zoom software (San Jose, USA).

For peer review only

Log data

Log data from Carer-eSupport will be collected after the test period to examine the ICs' activities and use patterns in the system, and to identify any indications of technical problems. Log data will show how Carer-eSupport works in practice and which components might be improved. They will also be used to improve the usability of Carer-eSupport.

Questionnaires

The ICs will be asked to complete questionnaires via the Portal (see below), before they are given access to Carer-eSupport. These questionnaires are planned to be used in an upcoming randomized controlled trial (RCT).

The Preparedness for caregiving scale [55, 56] is used to measure caregivers' perceived readiness to provide care in real time. It consists of eight items measuring preparedness to take care of the patient's needs, to obtain and set up services, to get necessary support from the healthcare system, and to manage IC-related stress. The scores are added to a total score ranging from 0 to 32, with a higher score reflecting better preparedness.

The caregiver's burden scale [57] will be used to assess IC burden. It consists of twenty-two items covering areas known to be especially important, such as IC health, psychological wellbeing, relationships, social network, and physical workload. Items are scored on a scale 1–4 (not at all, seldom, sometimes, often). A higher score reflects a higher burden.

The Short Form 36 version 2 (SF-36v2) is used to measure self-perceived health. It has been shown to have good psychometric properties [58]. It measures physical functioning, role limitations, bodily pain, general health, vitality, social functioning, and mental health. SF-36v2 encompasses a physical component and a mental component summary.

The Depression Anxiety Stress Scale-21 (DASS-21) [59] comprises three 7-item subscales measuring depression, anxiety, and stress. Each item is scored on a 4-point Likert scale, with higher scores indicating more symptoms. The DASS-21 has good psychometric properties [60].

The Multidimensional fatigue inventory (MFI-20)[61] will be used to measure fatigue. The questionnaire consists of 20 items that assess five dimensions of fatigue based on different modes of expression. A total score, ranging from 4 to 20, is calculated for each dimension by summation of the individual item scores. The Swedish version has been judged to possess good psychometric properties, in both groups of patients and healthy individuals [62].

For peer review only - http://bmjopen.bmj.com/site/about/guidelines.xhtml

Clinical and sociodemographic data will be collected from ICs, including age, gender, marital status, relationship to the patient, children living at home, level of education, psychological support, employment, economic situation, and computer skills. Data about patients' age, gender, diagnosis and tumour stage, and treatment (surgery, radio therapy, and pharmacological treatment) will be collected from medical records.

During the individual interviews (described above), the ICs will be asked about their experiences of the questionnaires, e.g., "What was your experience of answering the questionnaires?", "Did you think the questions were relevant to answer initially and after a month?", and "Did you find the questions easy to understand?"

Analysis

The quantitative data will be analyzed using descriptive statistics and the individual interviews using content analysis.

Changes to the Carer-eSupport will be made based on the results from the interviews and the analyses of log data. Additional tests will be conducted if large changes are made.

The research group

Our research group was formed in 2020 and consists of healthcare professionals from various fields and an expert in human-computer interaction. The members in the group has extensive experience of developing, testing, and evaluating internet-based complex interventions for ICs and patients [63, 64]. Our group includes expertise in the field of human-computer interaction and user-centred design,[65] especially eHealth applications, including gender aspects,[66] expertise regarding ICs in the cancer context,[67] and expertise in nursing and medical aspects of patients with HNC [68]. There is also experience of being an IC to an individual treated for HNC in the research group.

We are planning to form an expert panel with ICs to regularly advise the research group about the content and design of Carer-eSupport and how the project should be executed. In addition, we will invite an advisory board, consisting of researchers, managers in healthcare, politicians, and patient organization representatives to seminars, who can regularly provide their opinions on the project's progress and clinical relevance at seminars.

For peer review only

Discussion

The content of Carer-eSupport will be developed based on the experiences of ICs and healthcare professionals, scientific evidence, SCT, and UTAUT. This will add to the theoretical basis regarding ICs' needs. The goal is to develop a feasible and acceptable internet-based intervention to be evaluated in a forthcoming RCT regarding effects on self-reported preparedness for caregiving, caregiving burden, and IC health.

We will perform a solid targeted review, as recommended by O'Cathain et al.[60] instead of following a rigid systematic review methodology. Undertaking systematic reviews is not always necessary when developing a complex intervention, since there may be recent reviews available – nor is it always possible, given limited resources. However, this may be a limitation, as we may overlook valuable information from past research.

Based on MRC recommendations, [39] the feasibility and acceptability of the outcome measures planned to be used in the future RCT, will be evaluated in phase II. After this, the primary and secondary outcomes will be decided.

A thoroughly implementing of phases I and II and establishment of a panel of ICs and an advisory group, providing input on the implementation of the project increases the probability that Carer-eSupport can be implemented into healthcare, if the future RCT shows Carer-eSupport to be effective.

Carer-eSupport will be adapted to ICs experiencing a caregiver burden known to be high due to the advanced treatment given to the individuals they provide care to [6, 7]. Engaging stakeholders in the development phase [69, 70] is necessary to meet the ICs' needs. Stakeholders may find solutions that make a significant difference in the system development.

We are aware of the difficulties that may arise of conducting FGDs online. Some ICs may be unable to participate due to technical difficulties or not owning a computer. This may result in a lack of certain groups' experiences and needs. Also, patients with HNC make up a heterogenous group with differing needs and prognosis. We acknowledge this as a limitation. The intervention is tailored for ICs to individuals with HNC and it might be a challenge to meet all their needs and expectations.

We believe that ICs to individuals with HNC are motivated to learn caregiving skills [71]. Previous research has shown that ICs experience lack of preparedness. Carer-eSupport may

For peer review only - http://bmjopen.bmj.com/site/about/guidelines.xhtml

provide ICs with support and opportunities to acquire new skills and increase their self-efficacy for caregiving. SCT [42] provides a useful framework for understanding the ICs' behaviors and needs [43]. According to SCT, self-efficacy and behavioural capabilities, such as perceived preparedness, as well as environmental factors, may be important predictors of IC distress and could be useful for identifying those who might benefit most from Carer-eSupport. A strength is that we have complemented the theoretical framework with UTAUT,[44] to understand how each individual accepts technology. These theories serve as complementary tools for us to analyze the results and understand how to build Carer-eSupport.

Ethics and dissemination

All procedures have been approved by the Swedish Ethical Review Authority at Uppsala University (Dnr: 2020-04650). Informed consent will be obtained before enrolment of patients, their ICs, and healthcare staff. Confidentiality will be ensured by replacing names and personal identity numbers with codes in all materials. The coding lists will be kept separately from other research materials. All study data will be treated as confidential information and stored in safe archives, available only to members of the research group. Results will be disseminated in peer-reviewed journal publications. The feasibility testing (phase II) is registered at Clinicaltrials.gov (Identifier: NCT05028452).

Funding statement

This work was supported by the Swedish Research Council (grant number 2019-01231), the Swedish Cancer Society (grant number 20 1014 PjF), the Oncology Department Foundations Research Fund in Uppsala (grant number N/a) and the Swedish state under the agreement between the Swedish government and the county councils, the ALF- agreement (Uppsala county: ALF-941900).

Competing interests statement

None declared.

Authors' contributions: BJ conceived the study, contextualized Carer-eSupport, developed the protocol, is the grant holder of this study, and contributed critical input during drafting and finalization of the manuscript. All authors provided input on the design of Carer-eSupport. UL drafted and finalized the manuscript. YET contributed with critical input during drafting and finalization of the manuscript. ÅC, MC, and AA provided critical input to

For peer review only

finalize the protocol. GL and LvE contributed by critically commenting on and approving the final manuscript.

References

1. Hudson, P. and S. Payne, *The future of family caregiving: research, social policy and clinical practice.* Family Carers in Palliative Care: A guide for health and social care professionals, 2009: p. 2009.
2. Bray, F., et al., *Global cancer statistics 2018: GLOBOCAN estimates of incidence and mortality worldwide for 36 cancers in 185 countries.* CA: A Cancer Journal for Clinicians, 2018. **68**(6): p. 394-424.
3. Farnebo, L., et al., *Early death among head and neck cancer patients.* Current opinion in otolaryngology & head and neck surgery, 2016. **24**(2): p. 115-120.
4. Funk, G.F., L.H. Karnell, and A.J. Christensen, *Long-term health-related quality of life in survivors of head and neck cancer.* Arch Otolaryngol Head Neck Surg, 2012. **138**(2): p. 123-33.
5. Isaksson, J., et al., *Living an everyday life with head and neck cancer 2-2.5 years post-diagnosis - A qualitative prospective study of 56 patients.* Soc Sci Med, 2016. **154**: p. 54-61.
6. Ehrsson, Y.T., P. Fransson, and S. Einarsson, *Mapping Health-Related Quality of Life, Anxiety, and Depression in Patients with Head and Neck Cancer Diagnosed with*

For peer review only

Malnutrition Defined by GLIM. Nutrients, 2021. **13**(4): p. 1167.

7. Einarsson, S., G. Laurell, and Y. Tiblom Ehrsson, *Experiences and coping strategies related to food and eating up to two years after the termination of treatment in patients with head and neck cancer.* European journal of cancer care, 2019. **28**(2): p. e12964.
8. Ringash, J., et al., *Head and Neck Cancer Survivorship: Learning the Needs, Meeting the Needs.* Semin Radiat Oncol, 2018. **28**(1): p. 64-74.
9. Nund, R.L., et al., *Carers' experiences of dysphagia in people treated for head and neck cancer: a qualitative study.* Dysphagia, 2014. **29**(4): p. 450-8.
10. Patterson, J.M., et al., *Head and neck cancer and dysphagia; caring for carers.* Psychooncology, 2013. **22**(8): p. 1815-20.
11. Castellanos, E.H., et al., *Impact of patient symptoms and caregiving tasks on psychological distress in caregivers for head and neck cancer (HNC).* Psychooncology, 2019. **28**(3): p. 511-517.
12. Longacre, M.L., et al., *Psychological functioning of caregivers for head and neck cancer patients.* Oral Oncol, 2012. **48**(1): p. 18-25.
13. Lee, C.Y., et al., *Depression, anxiety, quality of life, and predictors of depressive disorders in caregivers of patients*

For peer review only

with head and neck cancer: A six-month follow-up study. J Psychosom Res, 2017. **100**: p. 29-34.

14. Schumacher, K.L., B.J. Stewart, and P.G. Archbold, *Conceptualization and measurement of doing family caregiving well.* Image J Nurs Sch, 1998. **30**(1): p. 63-9.
15. Given, B., et al., *Caregiver role strain.* Nursing Care of Older Adults diagnosis: Outcomes and Interventions. St. Louis, MO: Mosby, 2001: p. 679-95.
16. Braun, M., et al., *Hidden morbidity in cancer: spouse caregivers.* Journal of clinical oncology, 2007. **25**(30): p. 4829-4834.
17. Grunfeld, E., et al., *Family caregiver burden: results of a longitudinal study of breast cancer patients and their principal caregivers.* Cmaj, 2004. **170**(12): p. 1795-801.
18. Goldstein, N.E., et al., *Factors associated with caregiver burden among caregivers of terminally ill patients with cancer.* J Palliat Care, 2004. **20**(1): p. 38-43.
19. Ugalde, A., M. Krishnasamy, and P. Schofield, *Role recognition and changes to self-identity in family caregivers of people with advanced cancer: a qualitative study.* Support Care Cancer, 2012. **20**(6): p. 1175-81.
20. Carter, P.A., *Caregivers' descriptions of sleep changes and depressive symptoms.* Oncol Nurs Forum, 2002. **29**(9): p. 1277-83.

For peer review only - http://bmjopen.bmj.com/site/about/guidelines.xhtml

21. Carrera, P.M., H.M. Kantarjian, and V.S. Blinder, *The financial burden and distress of patients with cancer: Understanding and stepping-up action on the financial toxicity of cancer treatment.* CA Cancer J Clin, 2018. **68**(2): p. 153-165.
22. Kent, E.E. and J.N. Dionne-Odom, *Population-based profile of mental health and support service need among family caregivers of adults with cancer.* Journal of oncology practice, 2019. **15**(2): p. e122-e131.
23. Mason, N. and S. Hodgkin, *Preparedness for caregiving: A phenomenological study of the experiences of rural Australian family palliative carers.* Health & social care in the community, 2019. **27**(4): p. 926-935.
24. Kim, Y., H.R. Mitchell, and A. Ting, *Application of psychological theories on the role of gender in caregiving to psycho-oncology research.* Psychooncology, 2019. **28**(2): p. 228-254.
25. Ferrell, B. and E. Wittenberg, *A review of family caregiving intervention trials in oncology.* CA Cancer J Clin, 2017. **67**(4): p. 318-325.
26. Northouse, L.L., et al., *Interventions with family caregivers of cancer patients: meta-analysis of randomized trials.* CA Cancer J Clin, 2010. **60**(5): p. 317-39.
27. O'Toole, M.S., et al., *Cognitive behavioral therapies for informal caregivers of patients with cancer and cancer*

For peer review only - http://bmjopen.bmj.com/site/about/guidelines.xhtml

survivors: a systematic review and meta-analysis. Psychooncology, 2017. **26**(4): p. 428-437.

28. Applebaum, A.J. and W. Breitbart, *Care for the cancer caregiver: a systematic review.* Palliat Support Care, 2013. **11**(3): p. 231-52.
29. Belgacem, B., et al., *A caregiver educational program improves quality of life and burden for cancer patients and their caregivers: a randomised clinical trial.* Eur J Oncol Nurs, 2013. **17**(6): p. 870-6.
30. Chambers, S.K., et al., *A randomized trial comparing two low-intensity psychological interventions for distressed patients with cancer and their caregivers.* Oncol Nurs Forum, 2014. **41**(4): p. E256-66.
31. Chambers, S.K., et al., *A randomised controlled trial of a couples-based sexuality intervention for men with localised prostate cancer and their female partners.* Psychooncology, 2015. **24**(7): p. 748-56.
32. Treanor, C.J., *Psychosocial support interventions for cancer caregivers: reducing caregiver burden.* Curr Opin Support Palliat Care, 2020. **14**(3): p. 247-262.
33. Kaltenbaugh, D.J., et al., *Using Web-based interventions to support caregivers of patients with cancer: a systematic review.* Oncol Nurs Forum, 2015. **42**(2): p. 156-64.
34. Luo, X., et al., *A critical literature review of dyadic web-based interventions to support cancer patients and their*

For peer review only

caregivers, and directions for future research. Psychooncology, 2020. **29**(1): p. 38-48.

35. Ahmad, A., P. Mozelius, and K. Ahlin. *Testbed Requirements for Technology Enhanced Stroke Rehabilitation to Support Independent Living*. in *ICT4AWE*. 2019.
36. Freund, M., et al., *Effectiveness of information and communications technology interventions for stroke survivors and their support people: a systematic review.* Disability and Rehabilitation, 2021: p. 1-16.
37. Faruque, M., et al. *Feasibility of Digital Health Services for Educating the Community People Regarding Lifestyle Modification Combating Noncommunicable Diseases*. in *International Conference on Human-Computer Interaction*. 2019. Springer.
38. Ugalde, A., et al., *A systematic review of cancer caregiver interventions: Appraising the potential for implementation of evidence into practice.* Psycho-oncology, 2019. **28**(4): p. 687-701.
39. Craig, P., et al., *Developing and evaluating complex interventions: the new Medical Research Council guidance.* Bmj, 2008. **337**.
40. Firouzi, F., et al., *Ai-driven data monetization: The other face of data in iot-based smart and connected health.* IEEE Internet of Things Journal, 2020.

For peer review only

41. Cabestany, J., et al. *Artificial intelligence contribution to eHealth application*. in *2018 25th International Conference" Mixed Design of Integrated Circuits and System"(MIXDES)*. 2018. IEEE.
42. Schunk, D.H., *Social cognitive theory.* 2012.
43. Bandura, A., *Self-efficacy: toward a unifying theory of behavioral change.* Psychol Rev, 1977. **84**(2): p. 191-215.
44. Venkatesh, V., et al., *User acceptance of information technology: Toward a unified view.* MIS quarterly, 2003: p. 425-478.
45. Craig, P., et al., *Developing and evaluating complex interventions: the new Medical Research Council guidance.* Bmj, 2008. **337**: p. a1655.
46. Chan, A.-W., et al., *SPIRIT 2013 explanation and elaboration: guidance for protocols of clinical trials.* Bmj, 2013. **346**.
47. Creswell, J.W. and J.D. Creswell, *Research design: Qualitative, quantitative, and mixed methods approaches*. 2017: Sage publications.
48. Krueger, R.A. and M.A. Casey, *A practical guide for applied research.* A practical guide for applied research, 2000.
49. Lobe, B., D. Morgan, and K.A. Hoffman, *Qualitative Data Collection in an Era of Social Distancing.* International

For peer review only

Journal of Qualitative Methods, 2020. **19**: p. 1609406920937875.

50. Patton, M.Q., *Qualitative evaluation and research methods*. 1990: SAGE Publications, inc.

51. Lincoln, Y.S. and E.G. Guba, *But is it rigorous? Trustworthiness and authenticity in naturalistic evaluation.* New directions for program evaluation, 1986. **1986**(30): p. 73-84.

52. Sjöström, J., L. von Essen, and H. Grönqvist, *The origin and impact of ideals in eHealth research: experiences from the U-CARE research environment.* JMIR research protocols, 2014. **3**(2): p. e28.

53. The U-Care group. *Uppsala University Psychosocial Care Programme: U-CARE*. 2010 [cited 2016 Juni 15]; Available from: https://www.u-care.uu.se/u-care-portal.

54. Cocks, K. and D.J. Torgerson, *Sample size calculations for pilot randomized trials: a confidence interval approach.* Journal of Clinical Epidemiology, 2013. **66**(2): p. 197-201.

55. Henriksson, A., et al., *Adaptation and psychometric evaluation of the Preparedness for Caregiving Scale, Caregiver Competence Scale and Rewards of Caregiving Scale in a sample of Swedish family members of patients with life-threatening illness.* Palliat Med, 2012. **26**(7): p. 930-8.

For peer review only

56. Archbold, P.G., et al., *Mutuality and preparedness as predictors of caregiver role strain.* Research in nursing & health, 1990. **13**(6): p. 375-384.

57. Elmståhl, S., B. Malmberg, and L. Annerstedt, *Caregiver's burden of patients 3 years after stroke assessed by a novel caregiver burden scale.* Archives of physical medicine and rehabilitation, 1996. **77**(2): p. 177-182.

58. Taft, C., J. Karlsson, and M. Sullivan, *Performance of the Swedish SF-36 version 2.0.* Qual Life Res, 2004. **13**(1): p. 251-6.

59. Alfonsson, S., E. Wallin, and P. Maathz, *Factor structure and validity of the Depression, Anxiety and Stress Scale-21 in Swedish translation.* J Psychiatr Ment Health Nurs, 2017. **24**(2-3): p. 154-162.

60. Chiang, V.C.L., et al., *Fulfilling the psychological and information need of the family members of critically ill patients using interactive mobile technology: A randomised controlled trial.* Intensive Crit Care Nurs, 2017. **41**: p. 77-83.

61. Smets, E.M., et al., *The Multidimensional Fatigue Inventory (MFI) psychometric qualities of an instrument to assess fatigue.* J Psychosom Res, 1995. **39**(3): p. 315-25.

62. Hagelin, C.L., et al., *The psychometric properties of the Swedish Multidimensional Fatigue Inventory MFI-20 in*

For peer review only

four different populations. Acta Oncol, 2007. **46**(1): p. 97-104.

63. Cernvall, M., et al., *Twelve-Month Follow-Up of a Randomized Controlled Trial of Internet-Based Guided Self-Help for Parents of Children on Cancer Treatment.* J Med Internet Res, 2017. **19**(7): p. e273.
64. Wikman, A., et al., *Development of an Internet-Administered Cognitive Behavior Therapy Program (ENGAGE) for Parents of Children Previously Treated for Cancer: Participatory Action Research Approach.* J Med Internet Res, 2018. **20**(4): p. e133.
65. Gulliksen, J., et al., *Key principles for user-centred systems design.* Behaviour and Information Technology, 2003. **22**(6): p. 397-409.
66. Rexhepi, H., et al., *Cancer patients' attitudes and experiences of online access to their electronic medical records: A qualitative study.* Health informatics journal, 2018. **24**(2): p. 115-124.
67. Witkowski, Å. and M.E. Carlsson, *Support group programme for relatives of terminally ill cancer patients.* Supportive care in cancer, 2004. **12**(3): p. 168-175.
68. Stenhammar, C., et al., *Changes in intimate relationships following treatment for head and neck cancer-A qualitative study.* J Psychosoc Oncol, 2017. **35**(5): p. 614-630.

For peer review only - http://bmjopen.bmj.com/site/about/guidelines.xhtml

69. O'Cathain, A., et al., *Guidance on how to develop complex interventions to improve health and healthcare.* BMJ open, 2019. **9**(8): p. e029954.

70. Pandi-Perumal, S.R., et al., *Project Stakeholder Management in the Clinical Research Environment: How to Do it Right.* Frontiers in Psychiatry, 2015. **6**(71).

71. Badr, H., et al., *Unmet needs and relationship challenges of head and neck cancer patients and their spouses.* Journal of Psychosocial Oncology, 2016. **34**(4): p. 336-346.

For peer review only

For peer review only

For peer review only - http://bmjopen.bmj.com/site/about/guidelines.xhtml

Figure 1. Overview of the development phase and the feasibility testing phase of the intervention, Carer-eSupport, according to Medical Research Councils guidelines for complex intervention.

For peer review only

For peer review only - http://bmjopen.bmj.com/site/about/guidelines.xhtml

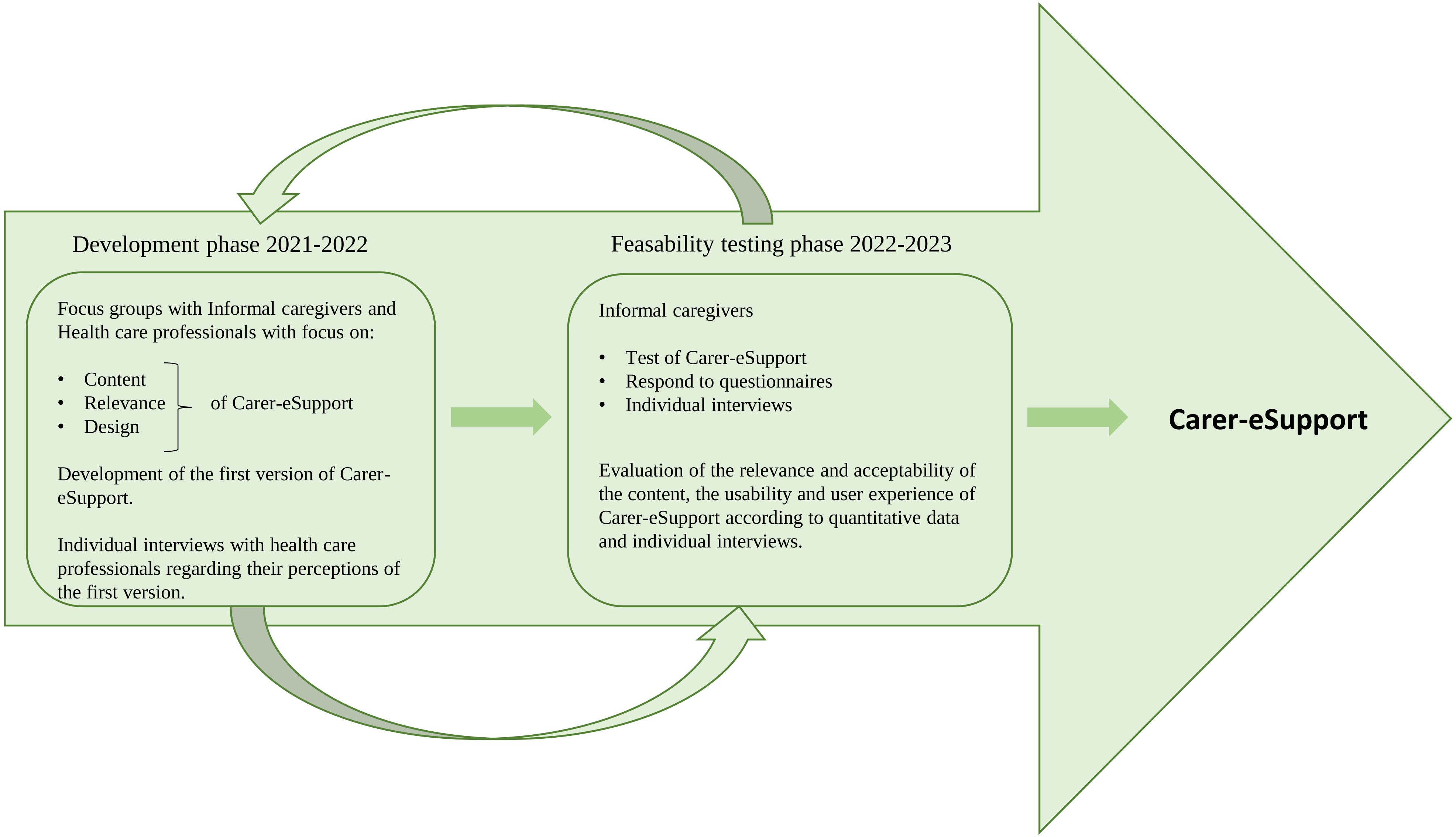
Development phase 2021-2022
Focus groups with Informal caregivers and Health care professionals with focus on:
• Content
• Relevance
• Design
of Carer-eSupport
Development of the first version of Carer-eSupport.
Individual interviews with health care professionals regarding their perceptions of the first version.
Feasability testing phase 2022-2023
Informal caregivers
• Test of Carer-eSupport
• Respond to questionnaires
• Individual interviews
Evaluation of the relevance and acceptability of the content, the usability and user experience of Carer-eSupport according to quantitative data and individual interviews.
Carer-eSupport

Reporting checklist for protocol of a clinical trial.

Based on the SPIRIT guidelines.

Instructions to authors

Complete this checklist by entering the page numbers from your manuscript where readers will find each of the items listed below.

Your article may not currently address all the items on the checklist. Please modify your text to include the missing information. If you are certain that an item does not apply, please write "n/a" and provide a short explanation.

Upload your completed checklist as an extra file when you submit to a journal.

In your methods section, say that you used the SPIRITreporting guidelines, and cite them as:

Chan A-W, Tetzlaff JM, Gøtzsche PC, Altman DG, Mann H, Berlin J, Dickersin K, Hróbjartsson A, Schulz KF, Parulekar WR, Krleža-Jerić K, Laupacis A, Moher D. SPIRIT 2013 Explanation and Elaboration: Guidance for protocols of clinical trials. BMJ. 2013;346:e7586

		Reporting Item	Page Number
**Administrative information**			
Title	#1	Descriptive title identifying the study design, population, interventions, and, if applicable, trial acronym	1

For peer review only

Trial registration	#2a	Trial identifier and registry name. If not yet registered, name of intended registry	1, 13<br><br>Clinicaltrials.gov. Feasibility study phase II.
Trial registration: data set	#2b	All items from the World Health Organization Trial Registration Data Set	N/a<br><br>The present study is feasibility study including informal caregivers and health professionals
Protocol version	#3	Date and version identifier	N/a. This is the only version.
Funding	#4	Sources and types of financial, material, and other support	13
Roles and responsibilities: contributorship	#5a	Names, affiliations, and roles of protocol contributors	Titel page, and p 14
Roles and responsibilities: sponsor contact information	#5b	Name and contact information for the trial sponsor	Titel page
Roles and responsibilities: sponsor and funder	#5c	Role of study sponsor and funders, if any, in study design; collection, management, analysis, and interpretation of data; writing of the report; and the decision to submit	N/a.

For peer review only

		the report for publication, including whether they will have ultimate authority over any of these activities	
Roles and responsibilities: committees	#5d	Composition, roles, and responsibilities of the coordinating centre, steering committee, endpoint adjudication committee, data management team, and other individuals or groups overseeing the trial, if applicable (see Item 21a for data monitoring committee)	N/a
**Introduction**			
Background and rationale	#6a	Description of research question and justification for undertaking the trial, including summary of relevant studies (published and unpublished) examining benefits and harms for each intervention	3
Background and rationale: choice of comparators	#6b	Explanation for choice of comparators	N/a. This is a feasibility study with an one arm feasibility study with a posttest design.
Objectives	#7	Specific objectives or hypotheses	5,6

For peer review only

Trial design	#8	Description of trial design including type of trial (eg, parallel group, crossover, factorial, single group), allocation ratio, and framework (eg, superiority, equivalence, non-inferiority, exploratory)	6
**Methods: Participants, interventions, and outcomes**			
Study setting	#9	Description of study settings (eg, community clinic, academic hospital) and list of countries where data will be collected. Reference to where list of study sites can be obtained	6
Eligibility criteria	#10	Inclusion and exclusion criteria for participants. If applicable, eligibility criteria for study centres and individuals who will perform the interventions (eg, surgeons, psychotherapists)	7,9
Interventions: description	#11a	Interventions for each group with sufficient detail to allow replication, including how and when they will be administered	8,9,10
Interventions: modifications	#11b	Criteria for discontinuing or modifying allocated interventions for a given trial	N/a

For peer review only

		participant (eg, drug dose change in response to harms, participant request, or improving / worsening disease)	
Interventions: adherance	#11c	Strategies to improve adherence to intervention protocols, and any procedures for monitoring adherence (eg, drug tablet return; laboratory tests)	Adherence will be assessed using log data from the portal and interviews.
Interventions: concomitant care	#11d	Relevant concomitant care and interventions that are permitted or prohibited during the trial	N/a
Outcomes	#12	Primary, secondary, and other outcomes, including the specific measurement variable (eg, systolic blood pressure), analysis metric (eg, change from baseline, final value, time to event), method of aggregation (eg, median, proportion), and time point for each outcome. Explanation of the clinical relevance of chosen efficacy and harm outcomes is strongly recommended	10
Participant timeline	#13	Time schedule of enrolment, interventions (including any run-ins and washouts), assessments, and visits for participants. A	Figure 1

For peer review only

		schematic diagram is highly recommended (see Figure)	
Sample size	#14	Estimated number of participants needed to achieve study objectives and how it was determined, including clinical and statistical assumptions supporting any sample size calculations	7
Recruitment	#15	Strategies for achieving adequate participant enrolment to reach target sample size	7, 9
**Methods: Assignment of interventions (for controlled trials)**			
Allocation: sequence generation	#16a	Method of generating the allocation sequence (eg, computer-generated random numbers), and list of any factors for stratification. To reduce predictability of a random sequence, details of any planned restriction (eg, blocking) should be provided in a separate document that is unavailable to those who enrol participants or assign interventions	N/a<br>This is a feasibility study with an uncontrolled feasibility study with a posttest design.

For peer review only

Allocation concealment mechanism	#16b	Mechanism of implementing the allocation sequence (eg, central telephone; sequentially numbered, opaque, sealed envelopes), describing any steps to conceal the sequence until interventions are assigned	N/a<br>This is a feasibility study with an uncontrolled feasibility study with a posttest design.
Allocation: implementation	#16c	Who will generate the allocation sequence, who will enrol participants, and who will assign participants to interventions	N/a
Blinding (masking)	#17a	Who will be blinded after assignment to interventions (eg, trial participants, care providers, outcome assessors, data analysts), and how	N/a
Blinding (masking): emergency unblinding	#17b	If blinded, circumstances under which unblinding is permissible, and procedure for revealing a participant's allocated intervention during the trial	N/a
**Methods: Data collection, management, and analysis**			
Data collection plan	#18a	Plans for assessment and collection of outcome, baseline, and other trial data,	10

For peer review only

		including any related processes to promote data quality (eg, duplicate measurements, training of assessors) and a description of study instruments (eg, questionnaires, laboratory tests) along with their reliability and validity, if known. Reference to where data collection forms can be found, if not in the protocol	
Data collection plan: retention	#18b	Plans to promote participant retention and complete follow-up, including list of any outcome data to be collected for participants who discontinue or deviate from intervention protocols	N/a<br>The aim of this feasibility study is to step by step thoroughly review all the processes and examine if intervention is relevant.
Data management	#19	Plans for data entry, coding, security, and storage, including any related processes to promote data quality (eg, double data entry; range checks for data values). Reference to where details of data management procedures can be found, if not in the protocol	8
Statistics: outcomes	#20a	Statistical methods for analysing primary and secondary outcomes. Reference to where other details of the statistical	11

For peer review only

		analysis plan can be found, if not in the protocol	
Statistics: additional analyses	#20b	Methods for any additional analyses (eg, subgroup and adjusted analyses)	8
Statistics: analysis population and missing data	#20c	Definition of analysis population relating to protocol non-adherence (eg, as randomised analysis), and any statistical methods to handle missing data (eg, multiple imputation)	N/a
**Methods: Monitoring**			
Data monitoring: formal committee	#21a	Composition of data monitoring committee (DMC); summary of its role and reporting structure; statement of whether it is independent from the sponsor and competing interests; and reference to where further details about its charter can be found, if not in the protocol. Alternatively, an explanation of why a DMC is not needed	N/a
Data monitoring: interim analysis	#21b	Description of any interim analyses and stopping guidelines, including who will have access to these interim results and	N/a

For peer review only

		make the final decision to terminate the trial	
Harms	#22	Plans for collecting, assessing, reporting, and managing solicited and spontaneously reported adverse events and other unintended effects of trial interventions or trial conduct	N/a
Auditing	#23	Frequency and procedures for auditing trial conduct, if any, and whether the process will be independent from investigators and the sponsor	N/a
**Ethics and dissemination**			
Research ethics approval	#24	Plans for seeking research ethics committee / institutional review board (REC / IRB) approval	13
Protocol amendments	#25	Plans for communicating important protocol modifications (eg, changes to eligibility criteria, outcomes, analyses) to relevant parties (eg, investigators, REC / IRBs, trial participants, trial registries, journals, regulators)	13
Consent or assent	#26a	Who will obtain informed consent or assent from potential trial participants or	6,13

For peer review only

		authorised surrogates, and how (see Item 32)	
Consent or assent: ancillary studies	#26b	Additional consent provisions for collection and use of participant data and biological specimens in ancillary studies, if applicable	N/a
Confidentiality	#27	How personal information about potential and enrolled participants will be collected, shared, and maintained in order to protect confidentiality before, during, and after the trial	13
Declaration of interests	#28	Financial and other competing interests for principal investigators for the overall trial and each study site	13
Data access	#29	Statement of who will have access to the final trial dataset, and disclosure of contractual agreements that limit such access for investigators	13,14<br>N/a. Data will only be handled in the research group.
Ancillary and post trial care	#30	Provisions, if any, for ancillary and post-trial care, and for compensation to those who suffer harm from trial participation	N/a
Dissemination policy: trial results	#31a	Plans for investigators and sponsor to communicate trial results to participants,	13

For peer review only

		healthcare professionals, the public, and other relevant groups (eg, via publication, reporting in results databases, or other data sharing arrangements), including any publication restrictions	
Dissemination policy: authorship	#31b	Authorship eligibility guidelines and any intended use of professional writers	14
Dissemination policy: reproducible research	#31c	Plans, if any, for granting public access to the full protocol, participant-level dataset, and statistical code	Present study protocol will be published with open access.

Appendices

Informed consent materials	#32	Model consent form and other related documentation given to participants and authorised surrogates	6<br>N/a<br>The participants confirm participation with a study specific informed consent.
Biological specimens	#33	Plans for collection, laboratory evaluation, and storage of biological specimens for genetic or molecular analysis in the current trial and for future use in ancillary studies, if applicable	N/a

For peer review only

None The SPIRIT Explanation and Elaboration paper is distributed under the terms of the Creative Commons Attribution License CC-BY-NC. This checklist can be completed online using https://www.goodreports.org/, a tool made by the EQUATOR Network in collaboration with Penelope.ai

For peer review only

BMJ Open

Internet based support for informal caregivers to individuals with head and neck cancer (Carer eSupport): A study protocol for the development and feasibility testing of a complex online intervention

Journal:	*BMJ Open*
Manuscript ID	bmjopen-2021-057442.R1
Article Type:	Protocol
Date Submitted by the Author:	25-Jan-2022
Complete List of Authors:	Langegård, Ulrica; Uppsala University, Department of Immunology, Genetics and Pathology, Experimental and Clinical Oncology<br>Cajander, Åsa; Uppsala Universitet Teknisk-naturvetenskapliga fakulteten, Department of Information Technology; Uppsala University<br>Carlsson, Maria; Uppsala University, Department of Public Health and Caring Sciences<br>von Essen, Louise; Uppsala University, Department of Women's and Children's Health, Healthcare Sciences and e-Health<br>Ahmad, Awais; Uppsala University, Department of Information Technology, Division of Visual Information and Interaction<br>Laurell, Göran; Uppsala University, Department of Surgical Sciences, Otorhinolaryngology and Head and Neck Surgery<br>Tiblom Ehrsson, Ylva; Uppsala University, Department of Surgical Sciences, Otorhinolaryngology and Head and Neck Surgery<br>Johansson, Birgitta; Uppsala University, Department of Immunology, Genetics and Pathology, Experimental and Clinical Oncology
**Primary Subject Heading**:	Oncology
Secondary Subject Heading:	Ear, nose and throat/otolaryngology, Nursing
Keywords:	ONCOLOGY, Protocols & guidelines < HEALTH SERVICES ADMINISTRATION & MANAGEMENT, RADIOTHERAPY, Adult radiotherapy < RADIOTHERAPY, QUALITATIVE RESEARCH, Head & neck tumours < ONCOLOGY

SCHOLARONE™
Manuscripts

Internet based support for informal caregivers to individuals with head and neck cancer (Carer eSupport): A study protocol for the development and feasibility testing of a complex online intervention

Ulrica Langegård, PhD, RN[1]; Åsa Cajander, PhD, Professor[2;] Maria Carlsson, PhD, Assoc Professor, RN[3]; Louise von Essen, PhD, Professor, Lic Psychologist[4]; Awais Ahmad, PhD student[2,] Göran Laurell, PhD, Professor, MD[5], Ylva Tiblom Ehrsson, Assoc Professor, RN[5*], Birgitta Johansson, PhD, Assoc Professor, RN[1*]

[1]Department of Immunology, Genetics and Patology, *Experimental and Clinical Oncology,* Uppsala University, Sweden.

[2] Department of Information Technology, Division of Visual Information and Interaction, Uppsala University, Sweden.

[3]Department of Public Health and Caring Sciences, Lifestyle and rehabilitation in long term illness, Uppsala University, Sweden.

[4] Department of Women's and Children's Health, Healthcare sciences and e-Health, Uppsala University, Sweden.

[5]Department of Surgical Sciences, Otorhinolaryngology and head and neck surgery, Uppsala University Sweden.

*equal contribution

Email addresses:

ulrica.langegard@igp.uu.se

asa.cajander@it.uu.se

maria.carlsson@pubcare.uu.se

louise-von.essen@kbh.uu.se

awais.ahmad@it.uu.se

goran.laurell@surgsci.uu.se

ylva.tiblom.ehrsson@surgsci.uu.se

birgitta.johansson@igp.uu.se

For peer review only

Corresponding author:

Ulrica Langegård, Department of immunology, genetics and patology, Uppsala University
Blod- och tumörsjukdomar, Administration
751 85 Uppsala
Telephone: +46 729-999020
Email: ulrica.langegard@igp.uu.se
ORCID id: orcid.org/0000-0001-8174-579X

Responsible for the trial

Birgitta Johansson, PhD, Assoc Professor, RN
Department of immunology, genetics and pathology, Uppsala University
Tel/Phone. +46 70425 09 12
ORCID: 0000-0001-6226-6849

Keywords: study protocol, development, feasibility testing, complex intervention, head and neck cancer, informal caregiver

Word count: 4531

For peer review only

Internet based support for informal caregivers to individuals with head and neck cancer (Carer eSupport): A study protocol for the development and feasibility testing of a complex online intervention

Abstract

Introduction

Informal caregivers (ICs) often play a vital role in the care and support of patients with cancer. The shift from inpatient to outpatient cancer care has resulted in a transition of care from professionals to ICs. This has increased the complexity of caregivers' responsibilities, which they may not be prepared for. There is a need to support ICs to prepare them for caregiving, decrease the caregiver burden, and prevent declining health. The main aims of this study are to develop a usable internet-based intervention to improve ICs' ability to support individuals with head and neck cancer and prevent IC health decline, identify the intervention's optimal design, and determine its feasibility and acceptability.

Methods and analysis

This is a multicentre study involving the ear, nose, and throat clinics and the oncology and radiotherapy clinics at three university hospitals. The study protocol comprises two phases, *development* and *feasibility testing*, using the Medical Research Council framework for developing a complex intervention. Carer eSupport will be based on the results from focus group discussions with ICs and healthcare professionals (planned N=6-8 in respectively group) and scientific evidence, the Social Cognitive Theory and the Theory of Acceptance and Use of Technology. Both qualitative and quantitative methods will be used. The goal is to develop a feasible and acceptable internet-based intervention to be evaluated in a forthcoming randomised controlled trial regarding effects on self-reported preparedness for caregiving, caregiving burden, and IC health.

Ethics and dissemination

All procedures have been approved by the Ethics committee at Uppsala University (Dnr: 2020-04650). Informed consent will be obtained before enrolment of patients, their ICs, and healthcare staff. The feasibility testing is registered at Clinicaltrials.gov (Identifier: NCT05028452). Findings will be disseminated in peer-reviewed journal publications.

For peer review only

For peer review only - http://bmjopen.bmj.com/site/about/guidelines.xhtml

Article Summary

Strengths and limitations of the study

- This protocol is the first to develop and test the feasibility of internet-based support for informal caregivers of individuals with head and neck cancer.
- Carer eSupport will be developed alongside informal caregivers and healthcare professionals, based on scientific evidence and established theories.
- The feasibility and usability of Carer eSupport will be tested using both quantitative and qualitative data.
- A solid targeted review will be performed, though this may result in loss of valuable research data in comparison to a systematic review.
- Carer eSupport will be provided via an established web portal, U-Care, which is not part of standard healthcare.

For peer review only

Internet based support for informal caregivers to individuals with head and neck cancer (Carer eSupport): A study protocol for the development and feasibility testing of a complex online intervention

Introduction

Informal caregivers (ICs) are crucial in the care and support of patients with cancer. An IC may be a relative, friend, or partner who provides (physical/emotional/practical) assistance to a patient.[1] The shift from inpatient to outpatient cancer care has resulted in a transition of care to ICs, increasing the complexity of their responsibilities, which they may not be prepared for. Thus, ICs may need support.

This study protocol describes the framing and design of a project, Carer eSupport, focusing on ICs to individuals with head and neck cancer (HNC). Carer eSupport is designed using the Medical Research Council (MRC) framework,[2] which includes four critical phases: (I) development, (II) feasibility testing, (III) evaluation, and (IV) implementation. This paper describes the first two phases for Carer eSupport (Figure 1). To ensure that the implementation of the intervention will proceed as planned, we will conduct a process evaluation during the feasibility phase. This will make it possible to identify internal (e.g., intervention design) and external factors (e.g., stakeholders, available resources) that may affect the primary and secondary outcomes, and the implementation process.[3, 4] Tentative models to be used in the process evaluation (planning in progress) is the logic model, Preced-Proceed planning model (PPM)[5] and the Information Systems Success Model,[6] to cover IT-specific questions.

Patients with head and neck cancer and their informal caregivers

Over 800,000 people worldwide were diagnosed with HNC in 2018.[7] HNC comprises several diagnoses with differing prognoses, and about 9.8% of those affected die within six months of diagnosis, due to tumour-, patient-, or treatment-related factors.[8] Many individuals with HNC experience symptom burden due to both the cancer and the treatments they receive, e.g., eating problems, pain, difficulty swallowing, and speech problems[9, 10] and physical and/or psychological distress.[11] This affects not only the patients, but also their ICs. Unmet needs have been identified among 60–70% of patients and a similar proportion of their ICs.[12] The ICs' responsibilities of symptoms management, including

For peer review only - http://bmjopen.bmj.com/site/about/guidelines.xhtml

dysphagia management, preparation of special meals and tube feeding, are time-consuming and may affect ICs' health negatively.[13, 14] Therefore, ICs may require support related to challenges along the trajectory, including skills training and psychological and social needs.[15] A recent study concerning ICs of individuals with HNC revealed that the perceived difficulty of caregiving tasks was a more crucial driver of psychological distress than the number of tasks or patient experiences of symptom clusters.[16] Lee et al.[17] observed that depressive disorder was the most prevalent psychiatric diagnosis in ICs to patients with HNC. Taken together, this indicates a need to develop support interventions for ICs of individuals with HNC.

For peer review only

A rationale for Carer eSupport

Preparedness and caregiver burden

Preparedness for caregiving can be defined as ICs' perceived capacity to provide physical, emotional, or practical care and manage the stresses of caregiving.[18] Lacking preparedness may increase caregiver burden, which can be described as a multidimensional biopsychosocial reaction resulting from an imbalance between care demands and a caregiver's care resources.[19] The caregiver burden may encompass physical, mental, and financial components and social isolation.[20-26] High perceived preparedness is associated with a lower caregiver burden and may prevent declining IC health[27] and increase patient care capacity.

Caregiver interventions

The effectiveness of interventions focusing on the ability to care for patients with cancer has been evaluated in several systematic reviews.[28-36] Northouse et al.[30] found that interventions for ICs' increased preparedness for caregiving, reduced caregiver burden and anxiety, and improved family relationships. Applebaum et al.[32] concluded that interventions combining different types of support, e.g., skill-building and psychoeducation, including support to cope with the illness, seemed to offer the greatest benefits. Ferrell et al.[29] showed that pain management skills are particularly important for caregivers, and suggested a focus on interventions focusing on general physical care and symptom assessment. A combination of interventions including instruction assessing side effects, techniques for assisting in pain management, and general training in problem-solving skills may increase ICs' preparedness for caregiving.[29] Aung et al.[37] suggested an educational programme to prepare caregivers for the complexity of their role. D'souza et al.[38] showed

For peer review only - http://bmjopen.bmj.com/site/about/guidelines.xhtml

that partners of patients with HNC, who followed a tailored education programme, reported higher satisfaction with information provision and less anxiety and depression than those who did not follow the programme. However, while various skill-based interventions have been evaluated, it remains unknown which approach has the best effects on ICs' preparedness for care and care burden.

Internet-based interventions

Internet-based health care interventions can act as a complement to standard care,[39] providing increased availability of information, education, and support, especially for people with limited possibilities to visit a clinic. In addition, internet-based interventions may be accessed anytime and when most needed. Thus, internet-based interventions need to be further studied, especially as regards effects on caregivers' preparedness for caregiving, physical health, and social and psychological outcomes.[40] There is a large variation in perceived meaningfulness of internet-based activities, highlighting the importance of adapting interventions to the needs and preferences of the intended end users. Willingness to use internet-based interventions depends on early and thorough involvement of end users in design and development.[41-43] Ugalde et al.[44] discussed the lack of actions to facilitate implementation in clinical settings. Crucial characteristics of a successful intervention are its relevance for the target groups and its potential to be effectively implemented in clinical care, provided that it is effective. Craig et al.[45] highlighted that early and continuous involvement of stakeholders, e.g., end users, in the development and feasibility testing of an intervention is necessary to achieve these goals. Thus, involvement of end users in the development and testing of Carer eSupport is pivotal, and a user-centred design methodology for systems development will be applied[46]. Further, artificial intelligence (AI) may create new opportunities for personalised and predictive healthcare systems[47, 48]. Carer eSupport can potentially be integrated with AI to provide ICs with relevant, accurate information, using users' experiences to increase ICs' preparedness for caregiving and decrease caregiver burden.

Theoretical framework

The Social Cognitive Theory (SCT)[49] is used as a theoretical framework for the development of Carer eSupport. The foundation for SCT is the significance of the interplay between behaviours and personal/social/environmental factors.[49] Self-efficacy is a core construct in SCT, conceptualised as a person's self-perceived capacity to perform in a

For peer review only - http://bmjopen.bmj.com/site/about/guidelines.xhtml

particular situation.[50] In the context of caring for patients with HNC, it can be assumed that ICs with high self-efficacy will perceive themselves as prepared to perform caregiving tasks, and therefore will be successful in doing so. SCT is supplemented with the well-established Unified Theory of Acceptance and Use of Technology (UTAUT) framework.[51] Technology acceptance models have been designed to predict behaviours and estimate acceptance and satisfaction in individuals using technology. The UTAUT model can also increase understanding of non-use of technology. UTAUT includes four relevant constructs: performance expectancy, effort expectancy, social influence, and facilitating conditions. In this study, the constructs are given the following meanings:

1. Performance expectancy refers to whether ICs believe that Carer eSupport can improve their preparedness for care.
2. Effort expectancy refers to the efforts ICs expect when using Carer eSupport.
3. Social influence refers to whether important people in an IC's surroundings believe the IC should use the intervention.
4. Facilitating conditions refers to enablers and barriers that may influence an IC's understanding of the ease or challenges of using Carer eSupport. These could be existing organizational or technical infrastructures that support use of the system.

In summary, the needs of ICs in providing support to individuals with HNC and factors of importance for their usage of and adherence to internet-based support remain to be explored. Systematic reviews suggest that interventions integrating multiple evidence-based modalities may increase ICs' preparedness for caregiving, decrease their caregiver burden, and improve their health. Interventions must fulfil ICs' needs so they can provide the best possible patient care that their resources permit. Thus, internet-based interventions for ICs should be developed, tested, and evaluated in collaboration with ICs and healthcare professionals (HCPs).

Aims

The aims are to develop a usable internet-based intervention to improve ICs' ability to support individuals with HNC and prevent IC health decline, identify the intervention's optimal design, and determine its feasibility and acceptability. An additional aim is to explore HCPs' perceptions of the content and design of Carer eSupport's usability from a clinical perspective.

For peer review only - http://bmjopen.bmj.com/site/about/guidelines.xhtml

Methods and analysis

Design

This study has two phases. Phase I encompasses development of Carer eSupport, including a theoretical framework, based on a literature review. Focus group discussion (FGDs) with ICs and HCPs will be performed. Development of the first version of Carer eSupport and interviews regarding HCPs' perceptions of this version will also be performed during this phase. Phase II includes individual interviews with ICs. Carer eSupport will be tested in a one-arm feasibility study with a post-test design. This protocol complies with the SPIRIT guidelines on writing protocols (see Additional file 1).[52]

Setting and procedure phases I and II

Participants are recruited at ear, nose, and throat and oncology and radiotherapy clinics at three Swedish university hospitals. Patients with HNC and their ICs will be identified by the HCPs responsible for patients care. ICs are designated by the patients, who must provide informed consent before their respective IC can be approached. HCPs who are asked to participate in the study will receive information about the study from a contact person at the hospital where they work, who will provide contact details to the research team (a project researcher). Next, ICs and HCPs will be contacted with more information about the study by the research team. They will be invited to participate and asked to provide informed consent.

Phase I - Development

Literature review and theoretical framework

A thorough review of the literature on the complexity of being an IC to a patient with cancer, in particular a patient with HNC, has been conducted and is presented in the introduction above. The literature review findings, SCT,[49] and UTAUT[51] will guide the development of a semi-structured interview guide for FGDs with ICs of patients with HNC.

Focus group discussions

Sample of informal caregivers

ICs are designated by patients; their eligibility is assessed as follows.

Inclusion criteria

- IC to a person with HNC about to start treatment, under treatment, or who has completed treatment not more than three months earlier.
- Age >18 years.

For peer review only - http://bmjopen.bmj.com/site/about/guidelines.xhtml

Exclusion criteria

- Confusion or dementia.
- Unable to understand, speak, and read Swedish.

People of different genders and ages, with experience of being an IC to an individual with HNC, is planned to be invited – to form one or two groups of 6–8 ICs at each of the three study sites.

Sample of healthcare professionals

The HCP groups will include people of different genders, ages, and professions, e.g., nurses, assistant nurses, physicians, psychologists, dietitians, speech therapists, and dental hygienists. At least one group of 6–8 HCPs at each of the three study sites is planned to be included. The inclusion criteria is having experience of caring for patients with HNC.

The semi-structured interviews

The FGDs with ICs[53, 54] will be divided into two parts. The first will focus on ICs' perceived experiences and needs. This may include knowledge and skills related to caring for individuals with HNC and support that could decrease the caregiver burden and increase ICs' preparedness for caregiving and ability to consider their own health. Self-care strategies and the need for psychosocial counselling will be explored. During the FGDs, open questions will be used to address ICs' experiences, e.g., 'What has been the major challenge for you as an IC to an individual with HNC?', 'What kind of support did you need as an IC to a person with HNC?', 'What support did you get that met your needs?', 'What support did you feel was lacking in your role as an IC?'.

The second part will explore the ICs' positive and negative experiences of internet-based support and how it should be designed to have high utility and provide an optimal user experience. It will also explore ICs' thoughts on integration of AI into Carer eSupport, for example, whether it's content should be automatically adapted depending on how an IC uses it. If necessary, additional individual interviews will be performed.

The FGDs with HCPs will be used to understand how Carer eSupport should be designed from a clinical perspective, and thereby facilitate potential implementation in standard care. HCPs' perceptions of ICs' needs for support concerning caregiving and wellbeing, and their views on how such support can be delivered via the internet in a clinical setting will be explored. The interview guide for HCPs contains questions such as 'What support do ICs of individuals treated for HNC need?', 'Which professions are needed in the support of the

For peer review only - http://bmjopen.bmj.com/site/about/guidelines.xhtml

ICs?', and 'How do you imagine that an internet-based support for ICs will be useful in clinical practice?'

Researchers (1 facilitator and 1 moderator) and a PhD student will conduct the FGDs and notes will be taken by the moderator, to capture participants' non-verbal expressions.

The FGDs will be conducted online using end-to-end encryption Zoom software with assurance that no unauthorised person can entrance the FGD. Zoom servers (San Jose, USA) in the European Union are used for Swedish Universities and comply with General Data Protection Regulations, GDPR[55] according to Swedish University regulations. The FGDs are expected to last 120–140 minutes, and will be audiotaped and transcribed.

Analysis of the focus group discussions

Transcripts and notes from FGDs will be analysed using thematic analysis, with an inductive and deductive research approach[56] to explore the underlying concepts and develop the intervention. By recording how codes will be developed from observations and ideas, the research team will use member checking and group debriefing to support the trustworthiness of the researcher's interpretations and analysis.[57]

Design and delivery of a first version of Carer eSupport

Carer eSupport will be provided via a web portal developed within the Uppsala University Psychosocial Care Program (U-CARE).[58, 59] The U-CARE-portal (Portal) has been developed and refined since 2010 and has been used in several previous projects. The Portal is designed for building and delivery of interventions and has several additional functions for instance for collecting informed consent online, performing randomization in studies, collecting self-report data, sending reminders at specific times, and logging user activity. The Portal uses a secure login process based on electronic identification, and all data is handled in a secure manner. Carer eSupport will be based on the best available scientific evidence and developed in parallel with the FGD analyses. The results of the thematic analysis will be concretised into supportive efforts (content in Carer eSupport) and used to build modules in the Portal, with additional support from ICs in the expert group (see below). A user-centred design methodology will be used in the development of Carer eSupport. The research team has extensive knowledge of development, testing, and evaluation of internet-based support, including usability and user experience. They will have the main responsibility for the development and contents of Carer eSupport, and will collaborate with other professionals to ensure that the entire spectrum of ICs' needs are considered.

For peer review only

The healthcare professionals' perceptions of the first version of Carer eSupport

The HCPs will be given access to the first version of Carer eSupport via the Portal for one month. Thereafter, individual interviews will be held with them regarding the features of Carer eSupport, its content and design, and if they encountered any technical problems. The interviews will be conducted online by the research team, using end-to-end encryption Zoom software (San Jose, USA).

Phase II - Feasibility testing

Phase II encompasses testing the relevance, acceptability, utility, and user experience of Carer eSupport.[2]

Sample of Informal caregivers

The recommendations of Cocks et al. on sample size in feasibility studies[60] would mean that approximately thirty ICs of patients with HNC need to be included to test the relevance and acceptability of the content, usability, and usefulness of Carer eSupport.

Inclusion criteria

- IC of an individual with HNC who is about to start, is undergoing, or has completed primary oncology treatment not more than one month earlier.
- Age >18 years.

Exclusion criteria

- Confusion or dementia.
- Unable to understand, speak, and read Swedish.

The test version of Carer eSupport

ICs will be given access to Carer eSupport for one month. The support is suggested to be delivered in the form of electronic live education for ICs and a library with written and audio-visual material. It will be possible to send messages and have conversations via a video link with HCPs, use discussion forums, and use digital rooms to exchange experiences with other ICs in a similar situation.

For peer review only

Feasibility testing

This feasibility study combines qualitative and quantitative methods. Carer eSupport will be considered feasible if ≥45% of eligible, approached ICs are recruited in participation, ≥50% of included ICs use the support (based on number of logins, use of educational materials according to log data and individual interviews), attrition is <30%, and ≥50% complete the questionnaires.[60] In addition, to be deemed feasible, Carer eSupport must be considered acceptable, relevant, and usable, based on qualitative data.

Qualitative data

Individual semi-structured interviews with ICs will be conducted after the test period. The interviews will include questions about the questionnaires, recruitment to the study and experiences of Carer eSupport (e.g., components, functions, layout, technical problems), in order to optimise its relevance and acceptability. The individual interviews will be conducted face to face or online by the researchers, using end-to-end encryption Zoom software (San Jose, USA).

Quantitative data

Log data from Carer eSupport will be collected after the test period to examine the ICs' activities and use patterns in the system, and to identify any indications of technical problems. This data will add knowledge on how Carer eSupport works in practice and which components might be improved and will also be used to improve the usability of Carer eSupport.

Questionnaires

The ICs will be asked to complete questionnaires via the Portal (see below) before they are given access to Carer eSupport. These questionnaires are planned to be used in a randomised controlled trial (RCT). However, questionnaires may be added or removed, depending on the outcome of Phase I and the feasibility testing.

The Preparedness for Caregiving scale[61, 62] is used to measure caregivers' perceived readiness to provide care in real time. It consists of eight items measuring preparedness to take care of the patient's needs, to obtain and set up services, to get necessary support from the healthcare system, and to manage IC-related stress. The scores are added to a total score ranging from 0 to 32, with a higher score reflecting better preparedness.

The Caregiver's Burden scale[63] will be used to assess IC burden. It consists of twenty-two items covering areas known to be especially important, such as IC health, psychological

For peer review only - http://bmjopen.bmj.com/site/about/guidelines.xhtml

wellbeing, relationships, social network, and physical workload. Items are scored on a 4-point Likert scale, with higher scores indicating more burden.

The Short Form 36 version 2 (SF-36v2) is used to measure self-perceived health. It has been shown to have good psychometric properties.[64] It measures physical functioning, role limitations, bodily pain, general health, vitality, social functioning, and mental health. SF-36v2 encompasses a physical component and a mental component summary.

The Depression Anxiety Stress Scale-21 (DASS-21)[65] comprises three 7-item subscales measuring depression, anxiety, and stress. Each item is scored on a 4-point Likert scale, with higher scores indicating more symptoms. The DASS-21 has good psychometric properties.[66]

The Multidimensional Fatigue Inventory (MFI-20)[67] will be used to measure fatigue. The questionnaire consists of 20 items that assess five dimensions of fatigue. A total score, ranging from 4 to 20, is calculated for each dimension by summation of the individual item scores. The Swedish version has good psychometric properties, for patients and healthy individuals.[68]

Clinical and sociodemographic data will be collected from ICs, including age, gender, marital status, relationship to the patient, children living at home, level of education, psychological support, employment, economic situation, and computer skills. Data about patients' age, gender, diagnosis and tumour stage, and treatment (surgery, radio therapy, and pharmacological treatment) will be collected from medical records.

During the individual interviews (described above), the ICs will be asked about their experiences of the questionnaires, e.g., 'What was your experience of answering the questionnaires?', 'Did you think the questions were relevant to answer initially and after a month?', 'Did you find the questions easy to understand' and 'Do you have thoughts and feelings that we haven't asked you about?'.

Analysis

The quantitative data, e.g., demographic data and log data, will be analysed using descriptive statistics. The individual interviews will be analysed using qualitative content analysis following the approach of Graneheim and Lundman.[69]

For peer review only

In phase II, depending on the results of the individual interviews and the analyses of the log data, it may be relevant to make changes in the Portal, e.g., alternative questionnaires, new content in the supportive efforts, or a changed layout.

Time plan

The preliminary timing of phase I is April 2021–March 2022, with phase II in March–November 2022.

The research team

Our research group was formed in 2020 and consists of HCPs from various fields and two experts in human-computer interaction. The members in the group have extensive experience of developing, testing, and evaluating internet-based complex interventions for ICs and patients.[70-73] Our group includes expertise in the field of human-computer interaction and user-centred design,[46] especially eHealth applications, including gender aspects,[74] expertise regarding ICs in the cancer context,[75] and expertise in nursing and medical aspects of patients with HNC.[76]

Patient and Public Involvement

One of the researcher in the research team has own experience of being an IC to an individual treated for HNC. In addition we will form an expert panel with ICs to regularly advise the research group about the content and design of Carer eSupport and how the project should be executed. Further, we will invite an advisory board, consisting of researchers, managers in healthcare, politicians, and patient organization representatives to seminars, who can regularly provide their opinions on the project's progress and clinical relevance at seminars.

Discussion

The content of Carer eSupport will be developed based on the experiences of ICs and HCPs, scientific evidence, SCT, and UTAUT. This will add to the theoretical basis regarding ICs' needs. The goal is to develop a feasible and acceptable internet-based intervention to be evaluated in a forthcoming RCT regarding effects on ICs' self-reported preparedness for caregiving, caregiving burden, and health.

We will perform a solid targeted review, as recommended by O'Cathain et al.[3] instead of following a systematic review methodology. Undertaking a systematic review is not always necessary when developing a complex intervention, since there may be recent reviews

For peer review only - http://bmjopen.bmj.com/site/about/guidelines.xhtml

available – nor is it always possible, given limited resources. However, this may be a limitation, as we may overlook valuable information from past research.

A thorough implementation of phases I and II and establishment of an expert panel consisting of ICs and an advisory group, providing input on the project implementation, will be crucial for the relevance of Carer eSupport. If the forthcoming RCT shows Carer eSupport to be effective regarding ICs' preparedness for caregiving and IC health, this will increase the probability that Carer eSupport can be implemented into healthcare.

Carer eSupport will be adapted to ICs who need to be prepared for caregiving at home due to the advanced treatment given to the individuals they provide care to.[9, 10] Engaging stakeholders, e.g., end users, in the development phase[3, 4] is therefore necessary to meet the ICs' needs. These stakeholders must have experience of caring for individuals with HNC at home, so they can find solutions that may make a difference for the ICs and identify and fill the gaps in relation to their needs. This is in line with the suggestions of Slev et al.[77]: that future studies should develop interventions and provide insight into the effects of eHealth on ICs in particular.

If a researcher does not perform a process evaluation when implementing a complex intervention, it is assumed that the implementation will proceed correctly, which rarely occurs. Therefore, we will perform a process evaluation. However, given the limited sample size, conclusions drawn should be considered with caution and supplemented with a process evaluation of the future RCT.

We are aware of the difficulties that may arise of conducting FGDs online. Some ICs may be unable to participate due to technical difficulties or not owning a computer. This may result in a lack of certain groups' experiences and needs. Also, patients with HNC make up a heterogenous group with differing needs and prognosis. We acknowledge this as a limitation. The intervention is adapted for ICs of individuals with HNC who have ongoing treatment or have been treated, and it might be a challenge to meet all their needs and expectations.

We believe that ICs of individuals with HNC are motivated to learn caregiving skills.[78] Previous research has shown that ICs experience lack of preparedness. Carer eSupport may provide ICs with support and opportunities to acquire new skills and increase their self-efficacy for caregiving. SCT[49] provides a useful framework for understanding the ICs' behaviours and needs.[50] According to SCT, self-efficacy and behavioural capabilities, such as perceived preparedness, as well as environmental factors, may be important predictors of

For peer review only

IC distress and could be useful for identifying those who might benefit most from Carer eSupport. A strength is that we have complemented the theoretical framework with UTAUT,[51] to understand how each individual accepts technology. These theories serve as complementary tools for us to analyse the results and understand how to build Carer eSupport.

Ethics and dissemination

All procedures have been approved by the Swedish Ethical Review Authority at Uppsala University (Dnr: 2020-04650). Informed consent will be obtained before enrolment of patients, their ICs, and HCPs. Confidentiality will be ensured by replacing names and personal identity numbers with codes in all materials. The coding lists will be kept separately from other research materials. All study data will be treated as confidential information and stored in safe archives, available only to members of the research group. Results will be disseminated in peer-reviewed journal publications. The feasibility testing (phase II) is registered at Clinicaltrials.gov (Identifier: NCT05028452).

Funding statement

This work was supported by the Swedish Research Council (grant number 2019-01231), the Swedish Cancer Society (grant number 20 1014 PjF), the Oncology Department Foundations Research Fund in Uppsala (grant number N/a) and the Swedish state under the agreement between the Swedish government and the county councils, the ALF- agreement (Uppsala county: ALF-941900).

Competing interests statement

None declared.

Authors' contributions: BJ conceived the study, contextualised Carer eSupport, developed the protocol, is the grant holder of this study, and contributed critical input during drafting and finalization of the manuscript. All authors provided input on the design of Carer eSupport. UL drafted and finalised the manuscript. YTE contributed with critical input during drafting and finalization of the manuscript. ÅC, MC, and AA provided critical input to finalise the protocol. GL and LvE contributed by critically commenting on and approving the final manuscript.

References

1. Hudson, P. and S. Payne, *The future of family caregiving: research, social policy and clinical practice.* Family Carers in Palliative Care: A guide for health and social care professionals, 2009: p. 2009.
2. Craig, P., et al., *Developing and evaluating complex interventions: the new Medical Research Council guidance.* Bmj, 2008. **337**: p. a1655.
3. O'Cathain, A., et al., *Guidance on how to develop complex interventions to improve health and healthcare.* BMJ open, 2019. **9**(8): p. e029954.

For peer review only

4. Pandi-Perumal, S.R., et al., *Project Stakeholder Management in the Clinical Research Environment: How to Do it Right.* Frontiers in Psychiatry, 2015. **6**(71).

5. Crosby, R. and S.M. Noar, *What is a planning model? An introduction to PRECEDE-PROCEED.* Journal of public health dentistry, 2011. **71**: p. S7-S15.

6. DeLone, W.H. and E.R. McLean, *The DeLone and McLean model of information systems success: a ten-year update.* Journal of management information systems, 2003. **19**(4): p. 9-30.

7. Bray, F., et al., *Global cancer statistics 2018: GLOBOCAN estimates of incidence and mortality worldwide for 36 cancers in 185 countries.* CA: A Cancer Journal for Clinicians, 2018. **68**(6): p. 394-424.

8. Farnebo, L., et al., *Early death among head and neck cancer patients.* Current opinion in otolaryngology & head and neck surgery, 2016. **24**(2): p. 115-120.

9. Ehrsson, Y.T., P. Fransson, and S. Einarsson, *Mapping Health-Related Quality of Life, Anxiety, and Depression in Patients with Head and Neck Cancer Diagnosed with Malnutrition Defined by GLIM.* Nutrients, 2021. **13**(4): p. 1167.

10. Einarsson, S., G. Laurell, and Y. Tiblom Ehrsson, *Experiences and coping strategies related to food and eating up to two years after the termination of treatment in*

For peer review only

patients with head and neck cancer. European journal of cancer care, 2019. **28**(2): p. e12964.

11. Wells, M., et al., *Distress, concerns and unmet needs in survivors of head and neck cancer: a cross-sectional survey.* European journal of cancer care, 2015. **24**(5): p. 748-760.
12. Ringash, J., et al., *Head and Neck Cancer Survivorship: Learning the Needs, Meeting the Needs.* Semin Radiat Oncol, 2018. **28**(1): p. 64-74.
13. Nund, R.L., et al., *Carers' experiences of dysphagia in people treated for head and neck cancer: a qualitative study.* Dysphagia, 2014. **29**(4): p. 450-8.
14. Patterson, J.M., et al., *Head and neck cancer and dysphagia; caring for carers.* Psychooncology, 2013. **22**(8): p. 1815-20.
15. Wang, T., S.R. Mazanec, and J.G. Voss. *Needs of Informal Caregivers of Patients With Head and Neck Cancer: A Systematic Review*. in *Oncology Nursing Forum*. 2021. Oncology Nursing Society.
16. Castellanos, E.H., et al., *Impact of patient symptoms and caregiving tasks on psychological distress in caregivers for head and neck cancer (HNC).* Psychooncology, 2019. **28**(3): p. 511-517.
17. Lee, C.Y., et al., *Depression, anxiety, quality of life, and predictors of depressive disorders in caregivers of patients*

For peer review only

with head and neck cancer: A six-month follow-up study. J Psychosom Res, 2017. **100**: p. 29-34.
18. Schumacher, K.L., B.J. Stewart, and P.G. Archbold, *Conceptualization and measurement of doing family caregiving well.* Image J Nurs Sch, 1998. **30**(1): p. 63-9.
19. Given, B., et al., *Caregiver role strain.* Nursing Care of Older Adults diagnosis: Outcomes and Interventions. St. Louis, MO: Mosby, 2001: p. 679-95.
20. Braun, M., et al., *Hidden morbidity in cancer: spouse caregivers.* Journal of clinical oncology, 2007. **25**(30): p. 4829-4834.
21. Grunfeld, E., et al., *Family caregiver burden: results of a longitudinal study of breast cancer patients and their principal caregivers.* Cmaj, 2004. **170**(12): p. 1795-801.
22. Goldstein, N.E., et al., *Factors associated with caregiver burden among caregivers of terminally ill patients with cancer.* J Palliat Care, 2004. **20**(1): p. 38-43.
23. Ugalde, A., M. Krishnasamy, and P. Schofield, *Role recognition and changes to self-identity in family caregivers of people with advanced cancer: a qualitative study.* Support Care Cancer, 2012. **20**(6): p. 1175-81.
24. Carter, P.A., *Caregivers' descriptions of sleep changes and depressive symptoms.* Oncol Nurs Forum, 2002. **29**(9): p. 1277-83.

For peer review only

25. Carrera, P.M., H.M. Kantarjian, and V.S. Blinder, *The financial burden and distress of patients with cancer: Understanding and stepping-up action on the financial toxicity of cancer treatment.* CA Cancer J Clin, 2018. **68**(2): p. 153-165.

26. Kent, E.E. and J.N. Dionne-Odom, *Population-based profile of mental health and support service need among family caregivers of adults with cancer.* Journal of oncology practice, 2019. **15**(2): p. e122-e131.

27. Mason, N. and S. Hodgkin, *Preparedness for caregiving: A phenomenological study of the experiences of rural Australian family palliative carers.* Health & social care in the community, 2019. **27**(4): p. 926-935.

28. Kim, Y., H.R. Mitchell, and A. Ting, *Application of psychological theories on the role of gender in caregiving to psycho-oncology research.* Psychooncology, 2019. **28**(2): p. 228-254.

29. Ferrell, B. and E. Wittenberg, *A review of family caregiving intervention trials in oncology.* CA Cancer J Clin, 2017. **67**(4): p. 318-325.

30. Northouse, L.L., et al., *Interventions with family caregivers of cancer patients: meta-analysis of randomized trials.* CA Cancer J Clin, 2010. **60**(5): p. 317-39.

31. O'Toole, M.S., et al., *Cognitive behavioral therapies for informal caregivers of patients with cancer and cancer*

For peer review only - http://bmjopen.bmj.com/site/about/guidelines.xhtml

survivors: a systematic review and meta-analysis. Psychooncology, 2017. **26**(4): p. 428-437.

32. Applebaum, A.J. and W. Breitbart, *Care for the cancer caregiver: a systematic review.* Palliat Support Care, 2013. **11**(3): p. 231-52.

33. Belgacem, B., et al., *A caregiver educational program improves quality of life and burden for cancer patients and their caregivers: a randomised clinical trial.* Eur J Oncol Nurs, 2013. **17**(6): p. 870-6.

34. Chambers, S.K., et al., *A randomized trial comparing two low-intensity psychological interventions for distressed patients with cancer and their caregivers.* Oncol Nurs Forum, 2014. **41**(4): p. E256-66.

35. Chambers, S.K., et al., *A randomised controlled trial of a couples-based sexuality intervention for men with localised prostate cancer and their female partners.* Psychooncology, 2015. **24**(7): p. 748-56.

36. Treanor, C.J., *Psychosocial support interventions for cancer caregivers: reducing caregiver burden.* Curr Opin Support Palliat Care, 2020. **14**(3): p. 247-262.

37. Aung, S.H.H., K. White, and J. Bloomfield, *The Experiences and the Needs of Caregivers of Patients With Head and Neck Cancer: An Integrative Review.* Cancer Nursing, 2021. **44**(6): p. E361-E373.

For peer review only

38. D'souza, V., et al., *Multimedia information intervention and its benefits in partners of the head and neck cancer patients.* European journal of cancer care, 2017. **26**(4): p. e12440.
39. Luo, X., et al., *A critical literature review of dyadic web-based interventions to support cancer patients and their caregivers, and directions for future research.* Psychooncology, 2020. **29**(1): p. 38-48.
40. Kaltenbaugh, D.J., et al., *Using Web-based interventions to support caregivers of patients with cancer: a systematic review.* Oncol Nurs Forum, 2015. **42**(2): p. 156-64.
41. Ahmad, A., P. Mozelius, and K. Ahlin. *Testbed Requirements for Technology Enhanced Stroke Rehabilitation to Support Independent Living*. in *ICT4AWE*. 2019.
42. Freund, M., et al., *Effectiveness of information and communications technology interventions for stroke survivors and their support people: a systematic review.* Disability and Rehabilitation, 2021: p. 1-16.
43. Faruque, M., et al. *Feasibility of Digital Health Services for Educating the Community People Regarding Lifestyle Modification Combating Noncommunicable Diseases*. in *International Conference on Human-Computer Interaction*. 2019. Springer.

For peer review only - http://bmjopen.bmj.com/site/about/guidelines.xhtml

44. Ugalde, A., et al., *A systematic review of cancer caregiver interventions: Appraising the potential for implementation of evidence into practice.* Psycho-oncology, 2019. **28**(4): p. 687-701.
45. Craig, P., et al., *Developing and evaluating complex interventions: the new Medical Research Council guidance.* Bmj, 2008. **337**.
46. Gulliksen, J., et al., *Key principles for user-centred systems design.* Behaviour and Information Technology, 2003. **22**(6): p. 397-409.
47. Firouzi, F., et al., *Ai-driven data monetization: The other face of data in iot-based smart and connected health.* IEEE Internet of Things Journal, 2020.
48. Cabestany, J., et al. *Artificial intelligence contribution to eHealth application*. in *2018 25th International Conference" Mixed Design of Integrated Circuits and System"(MIXDES)*. 2018. IEEE.
49. Schunk, D.H., *Social cognitive theory.* 2012.
50. Bandura, A., *Self-efficacy: toward a unifying theory of behavioral change.* Psychol Rev, 1977. **84**(2): p. 191-215.
51. Venkatesh, V., et al., *User acceptance of information technology: Toward a unified view.* MIS quarterly, 2003: p. 425-478.

For peer review only

52. Chan, A.-W., et al., *SPIRIT 2013 explanation and elaboration: guidance for protocols of clinical trials.* Bmj, 2013. **346**.
53. Creswell, J.W. and J.D. Creswell, *Research design: Qualitative, quantitative, and mixed methods approaches*. 2017: Sage publications.
54. Krueger, R.A. and M.A. Casey, *A practical guide for applied research.* A practical guide for applied research, 2000.
55. Lobe, B., D. Morgan, and K.A. Hoffman, *Qualitative Data Collection in an Era of Social Distancing.* International Journal of Qualitative Methods, 2020. **19**: p. 1609406920937875.
56. Braun, V. and V. Clarke, *Using thematic analysis in psychology.* Qualitative Research in Psychology, 2006. **3**(2): p. 77-101.
57. Lincoln, Y.S. and E.G. Guba, *But is it rigorous? Trustworthiness and authenticity in naturalistic evaluation.* New directions for program evaluation, 1986. **1986**(30): p. 73-84.
58. Sjöström, J., L. von Essen, and H. Grönqvist, *The origin and impact of ideals in eHealth research: experiences from the U-CARE research environment.* JMIR research protocols, 2014. **3**(2): p. e28.

For peer review only

59. The U-Care group. *Uppsala University Psychosocial Care Programme: U-CARE*. 2010 [cited 2016 Juni 15]; Available from: https://www.u-care.uu.se/u-care-portal.

60. Cocks, K. and D.J. Torgerson, *Sample size calculations for pilot randomized trials: a confidence interval approach.* Journal of Clinical Epidemiology, 2013. **66**(2): p. 197-201.

61. Henriksson, A., et al., *Adaptation and psychometric evaluation of the Preparedness for Caregiving Scale, Caregiver Competence Scale and Rewards of Caregiving Scale in a sample of Swedish family members of patients with life-threatening illness.* Palliat Med, 2012. **26**(7): p. 930-8.

62. Archbold, P.G., et al., *Mutuality and preparedness as predictors of caregiver role strain.* Research in nursing & health, 1990. **13**(6): p. 375-384.

63. Elmståhl, S., B. Malmberg, and L. Annerstedt, *Caregiver's burden of patients 3 years after stroke assessed by a novel caregiver burden scale.* Archives of physical medicine and rehabilitation, 1996. **77**(2): p. 177-182.

64. Taft, C., J. Karlsson, and M. Sullivan, *Performance of the Swedish SF-36 version 2.0.* Qual Life Res, 2004. **13**(1): p. 251-6.

65. Alfonsson, S., E. Wallin, and P. Maathz, *Factor structure and validity of the Depression, Anxiety and Stress Scale-*

For peer review only

21 in Swedish translation. J Psychiatr Ment Health Nurs, 2017. **24**(2-3): p. 154-162.

66. Chiang, V.C.L., et al., *Fulfilling the psychological and information need of the family members of critically ill patients using interactive mobile technology: A randomised controlled trial.* Intensive Crit Care Nurs, 2017. **41**: p. 77-83.
67. Smets, E.M., et al., *The Multidimensional Fatigue Inventory (MFI) psychometric qualities of an instrument to assess fatigue.* J Psychosom Res, 1995. **39**(3): p. 315-25.
68. Hagelin, C.L., et al., *The psychometric properties of the Swedish Multidimensional Fatigue Inventory MFI-20 in four different populations.* Acta Oncol, 2007. **46**(1): p. 97-104.
69. Graneheim, U.H. and B. Lundman, *Qualitative content analysis in nursing research: concepts, procedures and measures to achieve trustworthiness.* Nurse education today, 2004. **24**(2): p. 105-112.
70. Cernvall, M., et al., *Twelve-Month Follow-Up of a Randomized Controlled Trial of Internet-Based Guided Self-Help for Parents of Children on Cancer Treatment.* J Med Internet Res, 2017. **19**(7): p. e273.
71. Wikman, A., et al., *Development of an Internet-Administered Cognitive Behavior Therapy Program (ENGAGE) for Parents of Children Previously Treated for*

For peer review only - http://bmjopen.bmj.com/site/about/guidelines.xhtml

Cancer: Participatory Action Research Approach. J Med Internet Res, 2018. **20**(4): p. e133.

72. Woodford, J., et al., *Internet-administered cognitive behavioral therapy for common mental health difficulties in parents of children treated for cancer: intervention development and description study.* JMIR Formative Research, 2021. **5**(7): p. e22709.

73. Hauffman, A., et al., *Cocreated internet-based stepped care for individuals with cancer and concurrent symptoms of anxiety and depression: Results from the U-CARE AdultCan randomized controlled trial.* Psycho-Oncology, 2020. **29**(12): p. 2012-2018.

74. Rexhepi, H., et al., *Cancer patients' attitudes and experiences of online access to their electronic medical records: A qualitative study.* Health informatics journal, 2018. **24**(2): p. 115-124.

75. Witkowski, Å. and M.E. Carlsson, *Support group programme for relatives of terminally ill cancer patients.* Supportive care in cancer, 2004. **12**(3): p. 168-175.

76. Stenhammar, C., et al., *Changes in intimate relationships following treatment for head and neck cancer-A qualitative study.* J Psychosoc Oncol, 2017. **35**(5): p. 614-630.

77. Slev, V.N., et al., *Effects of eHealth for patients and informal caregivers confronted with cancer: a meta-review.*

For peer review only

International journal of medical informatics, 2016. **87**: p. 54-67.

78. Badr, H., et al., *Unmet needs and relationship challenges of head and neck cancer patients and their spouses.* Journal of Psychosocial Oncology, 2016. **34**(4): p. 336-346.

For peer review only

Figure 1. Overview of the development phase and the feasibility testing phase of the intervention, Carer eSupport, according to Medical Research Councils guidelines for complex intervention.

For peer review only

For peer review only - http://bmjopen.bmj.com/site/about/guidelines.xhtml

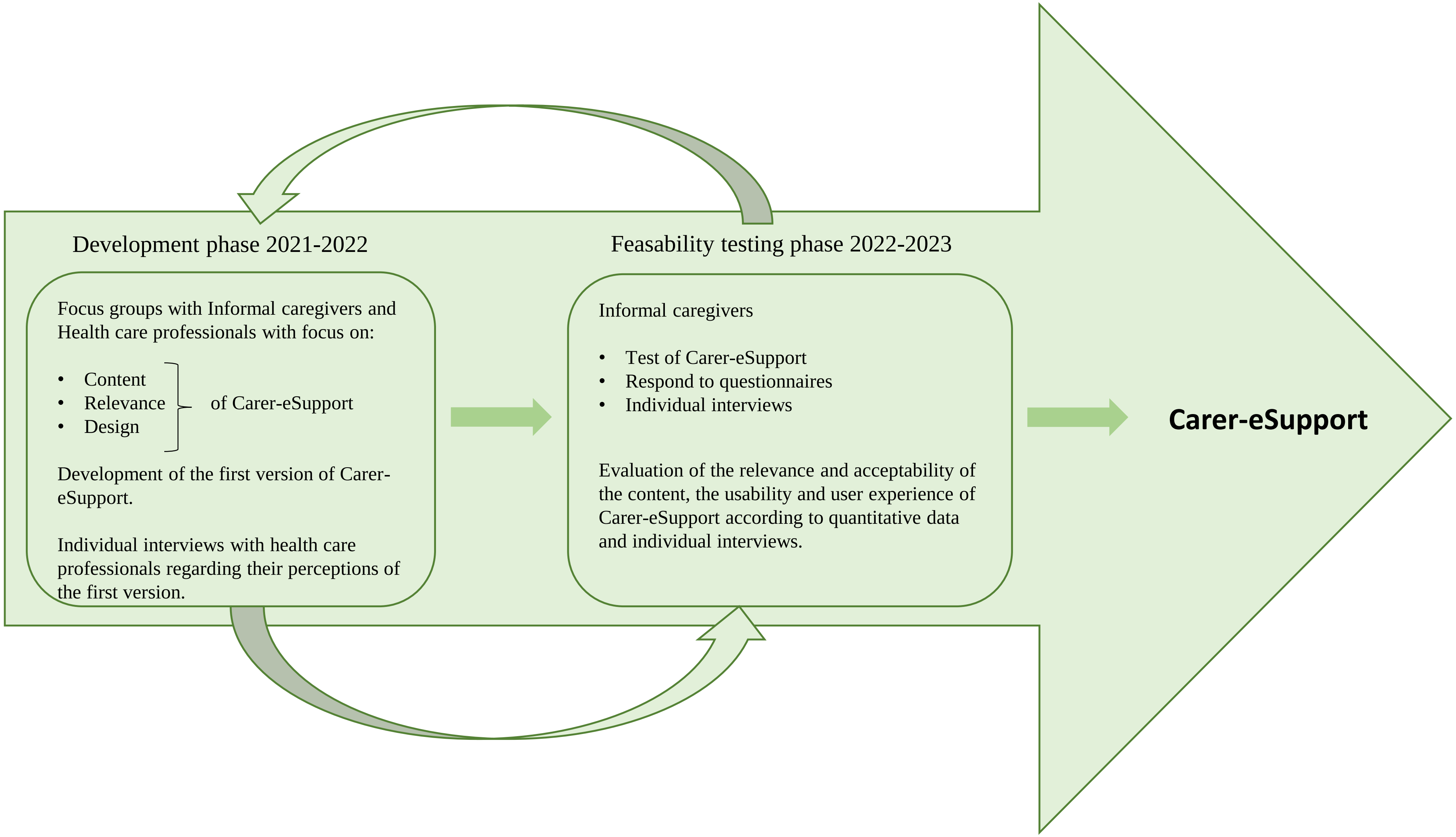
Development phase 2021-2022
Focus groups with Informal caregivers and Health care professionals with focus on:
• Content
• Relevance
• Design
of Carer-eSupport
Development of the first version of Carer-eSupport.
Individual interviews with health care professionals regarding their perceptions of the first version.
Feasability testing phase 2022-2023
Informal caregivers
• Test of Carer-eSupport
• Respond to questionnaires
• Individual interviews
Evaluation of the relevance and acceptability of the content, the usability and user experience of Carer-eSupport according to quantitative data and individual interviews.
Carer-eSupport

Reporting checklist for protocol of a clinical trial.

Based on the SPIRIT guidelines.

Instructions to authors

Complete this checklist by entering the page numbers from your manuscript where readers will find each of the items listed below.

Your article may not currently address all the items on the checklist. Please modify your text to include the missing information. If you are certain that an item does not apply, please write "n/a" and provide a short explanation.

Upload your completed checklist as an extra file when you submit to a journal.

In your methods section, say that you used the SPIRITreporting guidelines, and cite them as:

Chan A-W, Tetzlaff JM, Gøtzsche PC, Altman DG, Mann H, Berlin J, Dickersin K, Hróbjartsson A, Schulz KF, Parulekar WR, Krleža-Jerić K, Laupacis A, Moher D. SPIRIT 2013 Explanation and Elaboration: Guidance for protocols of clinical trials. BMJ. 2013;346:e7586

		Reporting Item	Page Number
**Administrative information**			
Title	#1	Descriptive title identifying the study design, population, interventions, and, if applicable, trial acronym	1

For peer review only

Trial registration	#2a	Trial identifier and registry name. If not yet registered, name of intended registry	1, 13<br>Clinicaltrials.gov.<br>Feasibility study phase II.
Trial registration: data set	#2b	All items from the World Health Organization Trial Registration Data Set	N/a<br>The present study is feasibility study including informal caregivers and health professionals
Protocol version	#3	Date and version identifier	N/a. This is the only version.
Funding	#4	Sources and types of financial, material, and other support	13
Roles and responsibilities: contributorship	#5a	Names, affiliations, and roles of protocol contributors	Titel page, and p 14
Roles and responsibilities: sponsor contact information	#5b	Name and contact information for the trial sponsor	Titel page
Roles and responsibilities: sponsor and funder	#5c	Role of study sponsor and funders, if any, in study design; collection, management, analysis, and interpretation of data; writing of the report; and the decision to submit	N/a.

For peer review only

		the report for publication, including whether they will have ultimate authority over any of these activities	
Roles and responsibilities: committees	#5d	Composition, roles, and responsibilities of the coordinating centre, steering committee, endpoint adjudication committee, data management team, and other individuals or groups overseeing the trial, if applicable (see Item 21a for data monitoring committee)	N/a
**Introduction**			
Background and rationale	#6a	Description of research question and justification for undertaking the trial, including summary of relevant studies (published and unpublished) examining benefits and harms for each intervention	3
Background and rationale: choice of comparators	#6b	Explanation for choice of comparators	N/a. This is a feasibility study with an one arm feasibility study with a posttest design.
Objectives	#7	Specific objectives or hypotheses	5,6

For peer review only

Trial design	#8	Description of trial design including type of trial (eg, parallel group, crossover, factorial, single group), allocation ratio, and framework (eg, superiority, equivalence, non-inferiority, exploratory)	6
**Methods: Participants, interventions, and outcomes**			
Study setting	#9	Description of study settings (eg, community clinic, academic hospital) and list of countries where data will be collected. Reference to where list of study sites can be obtained	6
Eligibility criteria	#10	Inclusion and exclusion criteria for participants. If applicable, eligibility criteria for study centres and individuals who will perform the interventions (eg, surgeons, psychotherapists)	7,9
Interventions: description	#11a	Interventions for each group with sufficient detail to allow replication, including how and when they will be administered	8,9,10
Interventions: modifications	#11b	Criteria for discontinuing or modifying allocated interventions for a given trial	N/a

For peer review only

		participant (eg, drug dose change in response to harms, participant request, or improving / worsening disease)	
Interventions: adherance	#11c	Strategies to improve adherence to intervention protocols, and any procedures for monitoring adherence (eg, drug tablet return; laboratory tests)	Adherence will be assessed using log data from the portal and interviews.
Interventions: concomitant care	#11d	Relevant concomitant care and interventions that are permitted or prohibited during the trial	N/a
Outcomes	#12	Primary, secondary, and other outcomes, including the specific measurement variable (eg, systolic blood pressure), analysis metric (eg, change from baseline, final value, time to event), method of aggregation (eg, median, proportion), and time point for each outcome. Explanation of the clinical relevance of chosen efficacy and harm outcomes is strongly recommended	10
Participant timeline	#13	Time schedule of enrolment, interventions (including any run-ins and washouts), assessments, and visits for participants. A	Figure 1

For peer review only

		schematic diagram is highly recommended (see Figure)	
Sample size	#14	Estimated number of participants needed to achieve study objectives and how it was determined, including clinical and statistical assumptions supporting any sample size calculations	7
Recruitment	#15	Strategies for achieving adequate participant enrolment to reach target sample size	7, 9
**Methods: Assignment of interventions (for controlled trials)**			
Allocation: sequence generation	#16a	Method of generating the allocation sequence (eg, computer-generated random numbers), and list of any factors for stratification. To reduce predictability of a random sequence, details of any planned restriction (eg, blocking) should be provided in a separate document that is unavailable to those who enrol participants or assign interventions	N/a<br>This is a feasibility study with an uncontrolled feasibility study with a posttest design.

For peer review only

Allocation concealment mechanism	#16b	Mechanism of implementing the allocation sequence (eg, central telephone; sequentially numbered, opaque, sealed envelopes), describing any steps to conceal the sequence until interventions are assigned	N/a<br>This is a feasibility study with an uncontrolled feasibility study with a posttest design.
Allocation: implementation	#16c	Who will generate the allocation sequence, who will enrol participants, and who will assign participants to interventions	N/a
Blinding (masking)	#17a	Who will be blinded after assignment to interventions (eg, trial participants, care providers, outcome assessors, data analysts), and how	N/a
Blinding (masking): emergency unblinding	#17b	If blinded, circumstances under which unblinding is permissible, and procedure for revealing a participant's allocated intervention during the trial	N/a
**Methods: Data collection, management, and analysis**			
Data collection plan	#18a	Plans for assessment and collection of outcome, baseline, and other trial data,	10

For peer review only

		including any related processes to promote data quality (eg, duplicate measurements, training of assessors) and a description of study instruments (eg, questionnaires, laboratory tests) along with their reliability and validity, if known. Reference to where data collection forms can be found, if not in the protocol	
Data collection plan: retention	#18b	Plans to promote participant retention and complete follow-up, including list of any outcome data to be collected for participants who discontinue or deviate from intervention protocols	N/a<br>The aim of this feasibility study is to step by step thoroughly review all the processes and examine if intervention is relevant.
Data management	#19	Plans for data entry, coding, security, and storage, including any related processes to promote data quality (eg, double data entry; range checks for data values). Reference to where details of data management procedures can be found, if not in the protocol	8
Statistics: outcomes	#20a	Statistical methods for analysing primary and secondary outcomes. Reference to where other details of the statistical	11

For peer review only

		analysis plan can be found, if not in the protocol	
Statistics: additional analyses	#20b	Methods for any additional analyses (eg, subgroup and adjusted analyses)	8
Statistics: analysis population and missing data	#20c	Definition of analysis population relating to protocol non-adherence (eg, as randomised analysis), and any statistical methods to handle missing data (eg, multiple imputation)	N/a
**Methods: Monitoring**			
Data monitoring: formal committee	#21a	Composition of data monitoring committee (DMC); summary of its role and reporting structure; statement of whether it is independent from the sponsor and competing interests; and reference to where further details about its charter can be found, if not in the protocol. Alternatively, an explanation of why a DMC is not needed	N/a
Data monitoring: interim analysis	#21b	Description of any interim analyses and stopping guidelines, including who will have access to these interim results and	N/a

For peer review only

		make the final decision to terminate the trial	
Harms	#22	Plans for collecting, assessing, reporting, and managing solicited and spontaneously reported adverse events and other unintended effects of trial interventions or trial conduct	N/a
Auditing	#23	Frequency and procedures for auditing trial conduct, if any, and whether the process will be independent from investigators and the sponsor	N/a
**Ethics and dissemination**			
Research ethics approval	#24	Plans for seeking research ethics committee / institutional review board (REC / IRB) approval	13
Protocol amendments	#25	Plans for communicating important protocol modifications (eg, changes to eligibility criteria, outcomes, analyses) to relevant parties (eg, investigators, REC / IRBs, trial participants, trial registries, journals, regulators)	13
Consent or assent	#26a	Who will obtain informed consent or assent from potential trial participants or	6,13

For peer review only

		authorised surrogates, and how (see Item 32)	
Consent or assent: ancillary studies	#26b	Additional consent provisions for collection and use of participant data and biological specimens in ancillary studies, if applicable	N/a
Confidentiality	#27	How personal information about potential and enrolled participants will be collected, shared, and maintained in order to protect confidentiality before, during, and after the trial	13
Declaration of interests	#28	Financial and other competing interests for principal investigators for the overall trial and each study site	13
Data access	#29	Statement of who will have access to the final trial dataset, and disclosure of contractual agreements that limit such access for investigators	13,14<br>N/a. Data will only be handled in the research group.
Ancillary and post trial care	#30	Provisions, if any, for ancillary and post-trial care, and for compensation to those who suffer harm from trial participation	N/a
Dissemination policy: trial results	#31a	Plans for investigators and sponsor to communicate trial results to participants,	13

For peer review only

		healthcare professionals, the public, and other relevant groups (eg, via publication, reporting in results databases, or other data sharing arrangements), including any publication restrictions	
Dissemination policy: authorship	#31b	Authorship eligibility guidelines and any intended use of professional writers	14
Dissemination policy: reproducible research	#31c	Plans, if any, for granting public access to the full protocol, participant-level dataset, and statistical code	Present study protocol will be published with open access.

Appendices

Informed consent materials	#32	Model consent form and other related documentation given to participants and authorised surrogates	6<br>N/a<br>The participants confirm participation with a study specific informed consent.
Biological specimens	#33	Plans for collection, laboratory evaluation, and storage of biological specimens for genetic or molecular analysis in the current trial and for future use in ancillary studies, if applicable	N/a

For peer review only

None The SPIRIT Explanation and Elaboration paper is distributed under the terms of the Creative Commons Attribution License CC-BY-NC. This checklist can be completed online using https://www.goodreports.org/, a tool made by the EQUATOR Network in collaboration with Penelope.ai

For peer review only

BMJ Open

Internet based support for informal caregivers to individuals with head and neck cancer (Carer eSupport): A study protocol for the development and feasibility testing of a complex online intervention

Journal:	*BMJ Open*
Manuscript ID	bmjopen-2021-057442.R2
Article Type:	Protocol
Date Submitted by the Author:	31-Mar-2022
Complete List of Authors:	Langegård, Ulrica; Uppsala University, Department of Immunology, Genetics and Pathology, Experimental and Clinical Oncology<br>Cajander, Åsa; Uppsala Universitet Teknisk-naturvetenskapliga fakulteten, Department of Information Technology; Uppsala University<br>Carlsson, Maria; Uppsala University, Department of Public Health and Caring Sciences<br>von Essen, Louise; Uppsala University, Department of Women's and Children's Health, Healthcare Sciences and e-Health<br>Ahmad, Awais; Uppsala University, Department of Information Technology, Division of Visual Information and Interaction<br>Laurell, Göran; Uppsala University, Department of Surgical Sciences, Otorhinolaryngology and Head and Neck Surgery<br>Tiblom Ehrsson, Ylva; Uppsala University, Department of Surgical Sciences, Otorhinolaryngology and Head and Neck Surgery<br>Johansson, Birgitta; Uppsala University, Department of Immunology, Genetics and Pathology, Experimental and Clinical Oncology
**Primary Subject Heading**:	Oncology
Secondary Subject Heading:	Ear, nose and throat/otolaryngology, Nursing
Keywords:	ONCOLOGY, Protocols & guidelines < HEALTH SERVICES ADMINISTRATION & MANAGEMENT, RADIOTHERAPY, Adult radiotherapy < RADIOTHERAPY, QUALITATIVE RESEARCH, Head & neck tumours < ONCOLOGY

SCHOLARONE™
Manuscripts

Internet based support for informal caregivers to individuals with head and neck cancer (Carer eSupport): A study protocol for the development and feasibility testing of a complex online intervention

Ulrica Langegård, PhD, RN[1]; Åsa Cajander, PhD, Professor[2]; Maria Carlsson, PhD, Assoc Professor, RN[3]; Louise von Essen, PhD, Professor, Lic Psychologist[4]; Awais Ahmad, PhD student[2], Göran Laurell, PhD, Professor, MD[5], Ylva Tiblom Ehrsson, Assoc Professor, RN[5]*, Birgitta Johansson, PhD, Assoc Professor, RN[1]*

[1]Department of Immunology, Genetics and Patology, *Experimental and Clinical Oncology,* Uppsala University, Sweden.

[2] Department of Information Technology, Division of Visual Information and Interaction, Uppsala University, Sweden.

[3]Department of Public Health and Caring Sciences, Lifestyle and rehabilitation in long term illness, Uppsala University, Sweden.

[4] Department of Women's and Children's Health, Healthcare sciences and e-Health, Uppsala University, Sweden.

[5]Department of Surgical Sciences, Otorhinolaryngology and head and neck surgery, Uppsala University Sweden.

*equal contribution

Email addresses:

ulrica.langegard@igp.uu.se

asa.cajander@it.uu.se

maria.carlsson@pubcare.uu.se

louise-von.essen@kbh.uu.se

awais.ahmad@it.uu.se

goran.laurell@surgsci.uu.se

ylva.tiblom.ehrsson@surgsci.uu.se

birgitta.johansson@igp.uu.se

For peer review only

Corresponding author:
Ulrica Langegård, Department of immunology, genetics and patology, Uppsala University
Blod- och tumörsjukdomar, Administration
751 85 Uppsala
Telephone: +46 729-999020
Email: ulrica.langegard@igp.uu.se
ORCID id: orcid.org/0000-0001-8174-579X

Responsible for the trial

Birgitta Johansson, PhD, Assoc Professor, RN
Department of immunology, genetics and pathology, Uppsala University
Tel/Phone. +46 70425 09 12
ORCID: 0000-0001-6226-6849

Keywords: study protocol, development, feasibility testing, complex intervention, head and neck cancer, informal caregiver

Word count: 4531

For peer review only - http://bmjopen.bmj.com/site/about/guidelines.xhtml

Internet based support for informal caregivers to individuals with head and neck cancer (Carer eSupport): A study protocol for the development and feasibility testing of a complex online intervention

Abstract

Introduction

It is strongly recommended that randomized controlled trials are preceded with an exploration of the needs of the target population and feasibility testing of the intervention. The present study protocol is set out to describe these steps in the development of a complex intervention.

The past decades' transition of care from inpatient to outpatient settings has increased the complexity of caregivers' responsibilities, which they may not be prepared for. There is a need to support informal caregivers (ICs) to prepare them for caregiving and decrease the caregiver burden. The main aim of this study is to describe the development of an internet-based intervention (Carer eSupport) to improve ICs' ability to support individuals with head and neck cancer (HNC) and to describe the testing of the feasibility and acceptability of Carer eSupport.

Methods and analysis

This is a multicentre study involving the ear, nose, and throat clinics and the oncology and radiotherapy clinics at three university hospitals. The study protocol comprises two phases, *development* and *feasibility testing*, using the Medical Research Council framework for developing a complex intervention. Carer eSupport will be based on the results from focus group discussions with ICs and healthcare professionals (planned for N=6-8 in respectively group) and scientific evidence, the Social Cognitive Theory and the Theory of Acceptance and Use of Technology. The feasibility testing will include 30 ICs who will have access to Carer eSupport for one month. The feasibility testing will be evaluated with a mixed method design.

Ethics and dissemination

All procedures have been approved by the Ethics committee at Uppsala University (Dnr: 2020-04650). Informed consent will be obtained before enrolment of patients, their ICs, and

For peer review only - http://bmjopen.bmj.com/site/about/guidelines.xhtml

healthcare staff. The feasibility testing is registered at Clinicaltrials.gov (Identifier: NCT05028452). Findings will be disseminated in peer-reviewed journal publications.

For peer review only

For peer review only - http://bmjopen.bmj.com/site/about/guidelines.xhtml

Article Summary

Strengths and limitations of the study

- Carer eSupport will be developed in collaboration with informal caregivers and healthcare professionals, and based on scientific evidence and established theories.
- The feasibility and acceptability of Carer eSupport will be tested using a mixed method.
- A thorough systematic review has not been conducted, this may result in loss of valuable research data in comparison to a systematic review.
- Carer eSupport will be provided via a research web portal, developed using a user centred design.

For peer review only

Internet based support for informal caregivers to individuals with head and neck cancer (Carer eSupport): A study protocol for the development and feasibility testing of a complex online intervention

Introduction

It is strongly recommended that a randomized controlled trial (RCT) is preceded by an exploration of the needs of the target population and feasibility testing of the intervention. [1] The present study protocol is set out to describe these steps in the development of a complex intervention that subsequently will be evaluated in a RCT.

Background

Informal caregivers (ICs) are crucial in the care and support of patients with cancer. An IC may be a relative, friend, or partner who provides (physical/emotional/practical) assistance to a patient.[2] The shift from inpatient to outpatient cancer care has resulted in a transition of care to ICs, increasing the complexity of their responsibilities, which they may not be prepared for.[3] Thus, ICs may need support.

This study protocol describes the design of a project, Carer eSupport, focusing on ICs to individuals with head and neck cancer (HNC). Carer eSupport is designed using the Medical Research Council (MRC) framework,[4] which includes four critical phases: (I) development, (II) feasibility testing, (III) evaluation, and (IV) implementation. This paper describes the first two phases for Carer eSupport (Figure 1). A forthcoming RCT will be conducted subsequently to evaluate the effects of Carer eSupport. To ensure a future implementation of the intervention, provided that Carer eSupport is effective, we will conduct a process evaluation.[5] This will make it possible to identify internal (e.g., intervention design) and external factors (e.g., stakeholders, available resources) that may affect outcomes, and the implementation process.[6, 7] Tentative models to be used in the process evaluation (planning in progress) is the logic model, Preced-Proceed planning model (PPM)[8] and the Information Systems Success Model,[9] to cover IT-specific questions.

Patients with head and neck cancer and their informal caregivers

Over 800,000 people worldwide were diagnosed with HNC in 2018.[10] Many individuals with HNC experience symptom burden due to both the cancer and the treatments they

For peer review only - http://bmjopen.bmj.com/site/about/guidelines.xhtml

receive, e.g., eating problems, pain, difficulty swallowing, and speech problems[11, 12] and physical and/or psychological distress.[13] This affects not only the patients, but also their ICs. Unmet needs have been identified among 60–70% of patients and a similar proportion of their ICs.[14] The ICs' responsibilities of symptoms management, including dysphagia management, preparation of special meals and tube feeding, are time-consuming and may affect ICs' health negatively.[15, 16] Therefore, ICs may require support related to challenges along the trajectory, including skills training and psychological and social needs.[17] A recent study concerning ICs of individuals with HNC revealed that the perceived difficulty of caregiving tasks was a more crucial driver of psychological distress than the number of tasks or patient experiences of symptom clusters.[18] Lee et al.[19] observed that depressive disorder was the most prevalent psychiatric diagnosis in ICs to patients with HNC. Taken together, this indicates a need to develop support interventions for ICs of individuals with HNC.

A rationale for Carer eSupport

Preparedness and caregiver burden

Preparedness for caregiving can be defined as ICs' perceived capacity to provide physical, emotional, or practical care and manage the stresses of caregiving.[20] Lacking preparedness may increase caregiver burden, which can be described as a multidimensional biopsychosocial reaction resulting from an imbalance between care demands and a caregiver's care resources.[21] The caregiver burden may encompass physical, mental [22, 23], and financial components[24], and social isolation.[25] High perceived preparedness is associated with a lower caregiver burden and may prevent declining IC health[26] and increase patient care capacity.

Caregiver interventions

The effectiveness of interventions focusing on the ability to care for patients with cancer has been evaluated in systematic reviews.[27-29] Northouse et al.[27] found that interventions for ICs' increased preparedness for caregiving, reduced caregiver burden and anxiety, and improved family relationships. Applebaum et al.[28] concluded that interventions combining different types of support, e.g., skill-building and psychoeducation, including support to cope with the illness, seemed to offer the greatest benefits. Ferrell et al.[29] showed that pain management skills are particularly important for caregivers, and suggested a focus on interventions focusing on general physical care and symptom assessment. A combination of

For peer review only

interventions including instruction assessing side effects, techniques for assisting in pain management, and general training in problem-solving skills may increase ICs' preparedness for caregiving.[29] Aung et al.[30] suggested an educational programme to prepare caregivers for the complexity of their role. D'souza et al.[31] showed that partners of patients with HNC, who followed a tailored education programme, reported higher satisfaction with information provision and less anxiety and depression than those who did not follow the programme. However, while various skill-based interventions have been evaluated, it remains unclear how interventions for ICs of patients with HNC may be designed to provide optimal support.

Internet-based interventions

Internet-based health care interventions can act as a complement to standard care,[32] providing increased availability of information, education, and support, especially for people with limited possibilities to visit a clinic. In addition, internet-based interventions may be accessed anytime and when most needed. Thus, internet-based interventions for ICs need to be further studied.[33] There is a large variation in perceived meaningfulness of internet-based activities, highlighting the importance of adapting interventions to the needs and preferences of the intended end users. Willingness to use internet-based interventions depends on early and thorough involvement of end users in design and development.[34-36] Ugalde et al.[37] discussed the lack of actions to facilitate implementation in clinical settings. Crucial characteristics of a successful intervention are its relevance for the target groups and its potential to be effectively implemented in clinical care, provided that it is effective. Craig et al.[1] highlighted that early and continuous involvement of stakeholders in the development and feasibility testing of an intervention is necessary to achieve these goals. Thus, ICs will be involved in the entire process of the development and testing of Carer eSupport. In addition, healthcare professionals (HCPs) perceptions of the intervention will be explored to facilitate an eventual implementation in clinical care after the forthcoming RCT has been conducted. Further, artificial intelligence (AI) may create new opportunities for personalised and predictive healthcare systems[38, 39]. Carer eSupport can potentially be integrated with AI to provide ICs with relevant, accurate information, using users' experiences to increase ICs' preparedness for caregiving and decrease caregiver burden.

For peer review only

Theoretical framework

The Social Cognitive Theory (SCT)[40] is used as a theoretical framework for the development of Carer eSupport. The foundation for SCT is the significance of the interplay between behaviours and personal/social/environmental factors.[40] Self-efficacy is a core construct in SCT, conceptualised as a person's self-perceived capacity to perform in a particular situation.[41] In the context of caring for patients with HNC, it can be assumed that ICs with high self-efficacy will perceive themselves as prepared to perform caregiving tasks, and therefore will be successful in doing so. SCT is supplemented with the well-established Unified Theory of Acceptance and Use of Technology (UTAUT) framework.[42] Technology acceptance models have been designed to predict behaviours and estimate acceptance and satisfaction in individuals using technology. The UTAUT model can also increase understanding of non-use of technology. UTAUT includes four relevant constructs: performance expectancy, effort expectancy, social influence, and facilitating conditions. In this study, the constructs are given the following meanings:

1. Performance expectancy refers to whether ICs believe that Carer eSupport can improve their preparedness for care.
2. Effort expectancy refers to the efforts ICs expect when using Carer eSupport.
3. Social influence refers to whether important people in an IC's surroundings believe the IC should use the intervention.
4. Facilitating conditions refers to enablers and barriers that may influence an IC's understanding of the ease or challenges of using Carer eSupport. These could be existing organizational or technical infrastructures that support use of the system.

In summary, the needs of ICs in providing support to individuals with HNC and factors of importance for their usage of and adherence to internet-based support remain to be explored. Systematic reviews suggest that interventions integrating multiple evidence-based modalities may increase ICs' preparedness for caregiving, decrease their caregiver burden, and improve their health. Interventions must fulfil ICs' needs so they can provide the best possible patient care that their resources permit. Thus, internet-based interventions for ICs should be developed, tested, and evaluated in collaboration with ICs and HCPs.

Aims

The main aim is to describe the development of an internet-based intervention (Carer eSupport) to improve ICs' ability to support individuals with HNC, and the testing of the

For peer review only

Carer eSupport's feasibility and acceptability. An additional aim is to explore HCPs' perceptions of the content and design of Carer eSupport from a clinical perspective.

Methods and analysis

Design

This protocol describes the planning of two phases. Phase I encompasses the development of Carer eSupport, based on focus groups discussion (FGDs) with ICs and HCPs, a theoretical framework, and a literature review. ICs' and HCPs' perceptions of early versions of Carer eSupport will also be collected. Phase II is a one-arm feasibility study with a post-test design and includes individual interviews with ICs who will have had access to the first version of Carer eSupport for one month. This protocol complies with the SPIRIT guidelines on writing protocols (see Additional file 1).[43]

Setting and procedure phases I and II

Participants are recruited at ear, nose, and throat and oncology and radiotherapy clinics at three Swedish university hospitals. Patients with HNC and their ICs will be identified by the HCPs responsible for patients care. ICs are designated by the patients, who must provide informed consent before their respective IC can be approached. HCPs who are asked to participate in the study will receive information about the study from a contact person at the hospital where they work, who will provide contact details to the research team (a project researcher). Next, ICs and HCPs will be contacted with more information about the study by the research team. They will be invited to participate and asked to provide informed consent.

Phase I - Development

Literature review and theoretical framework

A review of the literature on the complexity of being an IC to a patient with cancer, in particular a patient with HNC, has been conducted and is presented in the introduction above. The literature review findings, SCT,[40] and UTAUT[42] will guide the development of a semi-structured interview guide for FGDs with ICs of patients with HNC.

Focus group discussions

Sample of informal caregivers

ICs are designated by patients; their eligibility is assessed as follows.

Inclusion criteria

For Peer review only

- IC to a person with HNC about to start treatment, under treatment, or who has completed treatment not more than three months earlier.
- Age >18 years.

Exclusion criteria

- Confusion or dementia.
- Unable to understand, speak, and read Swedish.

People of different genders and ages, with experience of being an IC to an individual with HNC, is planned to be invited – to form one or two groups of 6–8 ICs at each of the three study sites.

Sample of healthcare professionals

HCPs are included to receive clinicians' aspects of internet-based support for ICs to facilitate an eventual implementation after the forthcoming RCT. The HCP groups will include people of different genders, ages, and professions, e.g., nurses, assistant nurses, physicians, psychologists, dietitians, speech therapists, and dental hygienists. At least one group of 6–8 HCPs at each of the three study sites is planned to be included. The inclusion criteria is having experience of caring for patients with HNC.

The semi-structured interviews

The FGDs with ICs[44, 45] will be divided into two parts. The first will focus on ICs' perceived experiences and needs. This may include knowledge and skills related to caring for individuals with HNC and support that could decrease the caregiver burden and increase ICs' preparedness for caregiving and ability to consider their own health. Self-care strategies and the need for psychosocial counselling will be explored. During the FGDs, open questions will be used to address ICs' experiences, e.g., 'What has been the major challenge for you as an IC to an individual with HNC?', 'What kind of support did you need as an IC to a person with HNC?', 'What support did you get that met your needs?', 'What support did you feel was lacking in your role as an IC?'.

The second part will explore the ICs' positive and negative experiences of internet-based support and how it should be designed to have high utility and provide an optimal user experience. It will also explore ICs' thoughts on integration of AI into Carer eSupport, for example, whether it's content should be automatically adapted depending on how an IC uses it. If necessary, additional individual interviews will be performed.

For peer review only

The FGDs with HCPs will be used to understand how Carer eSupport should be designed from a clinical perspective, and thereby facilitate potential implementation in standard care. HCPs' perceptions of ICs' needs for support concerning caregiving and wellbeing, and their views on how such support can be delivered via the internet in a clinical setting will be explored. The interview guide for HCPs contains questions such as 'What support do ICs of individuals treated for HNC need?', 'Which professions are needed in the support of the ICs?', and 'How do you imagine that an internet-based support for ICs will be useful in clinical practice?'

Researchers (1 facilitator and 1 moderator) and a PhD student will conduct the FGDs and notes will be taken by the moderator, to capture participants' non-verbal expressions.

The FGDs will be conducted online using end-to-end encryption Zoom software with assurance that no unauthorised person can entrance the FGD. Zoom servers (San Jose, USA) in the European Union are used for Swedish Universities and comply with General Data Protection Regulations, GDPR[46] according to Swedish University regulations. The FGDs are expected to last 120–140 minutes, and will be audiotaped and transcribed.

Analysis of the focus group discussions

Transcripts and notes from FGDs will be analysed using thematic analysis, research approach[47] to explore the underlying concepts and develop the intervention. By recording how codes will be developed from observations and ideas, the research team will use member checking and group debriefing during and after FDGs to support the trustworthiness of the researcher's interpretations and analysis.[48]

Design and delivery of a first version of Carer eSupport

Carer eSupport will be provided via a web portal developed within the Uppsala University Psychosocial Care Program (U-CARE).[49, 50] The U-CARE-portal (Portal) has been developed and refined since 2010 and has been used in several previous projects. The Portal is designed for building and delivery of interventions and include features for randomization of study participants, collecting self-report data, sending reminders at specific times, and logging user activity. The Portal uses a secure login process based on electronic identification, and all data is handled in a secure manner. A user-centred design methodology will be used in the development of the specific features of Carer eSupport. This implies that the U-CARE system developers take into account opinions and feedback from an IC expert group (see below) regarding the features of Carer eSupport repeatedly during the

For peer review only

development process.[51] The content of Carer eSupport will be based on the best available scientific evidence and the results of the FGD. The IC expert group will provide opinions regarding the content at several meetings throughout the development of the content. The research team has extensive knowledge of development, testing, and evaluation of internet-based support, including usability and user experience. They will have the main responsibility for the contents and the development of Carer eSupport, in collaboration with the IC expert group and HCP experts. HCPs ensure that the entire spectrum of ICs' needs is considered.

The healthcare professionals' perceptions of an early version of Carer eSupport

The HCPs will be given access to an early version of Carer eSupport for one month. This is done to receive their view about how Carer eSupport may be improved before the feasibility testing. Individual interviews will be held with them regarding the features of Carer eSupport, its content and design, and if they encountered any technical problems. The interviews will be conducted online by the research team, using end-to-end encryption Zoom software (San Jose, USA).

Phase II - Feasibility testing

Phase II encompasses testing the relevance, acceptability, and user experience of Carer eSupport.[4]

Sample of Informal caregivers

The recommendations of Cocks et al. on sample size in feasibility studies[52] would mean that approximately thirty ICs of patients with HNC need to be included. The feasibility and acceptability of Carer eSupport, will be evaluated to increase the robustness of the forthcoming RCT.

Inclusion criteria

- IC of an individual with HNC who is about to start, is undergoing, or has completed primary oncology treatment not more than one month earlier.
- Age >18 years.

Exclusion criteria

- Confusion or dementia.
- Unable to understand, speak, and read Swedish.

For peer review only - http://bmjopen.bmj.com/site/about/guidelines.xhtml

The test version of Carer eSupport

This is a tentative description of the first version of Carer eSupport which still is to be developed during Phase I. ICs will be given access to Carer eSupport for one month. The support may be delivered in the form of electronic live education for ICs and a library with written and audio-visual material. It may be possible to send messages and have conversations via a video link with HCPs, use discussion forums, and use digital rooms to exchange experiences with other ICs in a similar situation.

Feasibility testing

Explanation and Justification of Method

This feasibility study will use a mixed-method design, including analysis and integration of qualitative and quantitative data.[44] In the qualitative approach, individual semi-structured interviews with ICs will be conducted after the test period. The interviews will explore the ICs' experiences of Carer eSupport (e.g., the content, functions, layout, and technical problems). Their experiences with the questionnaires, the recruitment to the study will also be explored. The individual interviews will be conducted face to face or online by the researchers, using end-to-end encryption Zoom software (San Jose, USA). In the quantitative approach, log data from Carer eSupport will be collected after the test period to examine the ICs' activities and use patterns in the system, and to identify any indications of technical problems. This data will provide knowledge about how Carer eSupport, the recruitment procedures, and the choice of outcomes may be improved to increase the robustness of the forthcoming RCT. Depending on the results of the feasibility study it may be relevant to make changes in the Carer eSuppoprt.

Carer eSupport will be considered feasible if ≥45% of eligible, approached ICs are recruited in participation, ≥50% of included ICs use the support (based on number of logins, use of educational materials according to log data and individual interviews), attrition is <30%, and ≥50% complete the questionnaires.[52] In addition, to be deemed feasible, Carer eSupport must be considered acceptable, relevant, and usable, based on qualitative data.

Questionnaires

The ICs will be asked to complete questionnaires via the Portal (see below) before they are given access to Carer eSupport. These questionnaires are planned to be used in a randomised controlled trial (RCT). However, questionnaires may be added or removed, depending on the outcome of Phase I and the feasibility testing.

For peer review only

The Preparedness for Caregiving scale[53, 54] is used to measure caregivers' perceived readiness to provide care in real time. It consists of eight items measuring preparedness to take care of the patient's needs, to obtain and set up services, to get necessary support from the healthcare system, and to manage IC-related stress. The scores are added to a total score ranging from 0 to 32, with a higher score reflecting better preparedness.

The Caregiver's Burden scale[55] will be used to assess IC burden. It consists of twenty-two items covering areas known to be especially important, such as IC health, psychological wellbeing, relationships, social network, and physical workload. Items are scored on a 4-point Likert scale, with higher scores indicating more burden.

The Short Form 36 version 2 (SF-36v2) is used to measure self-perceived health. It has been shown to have good psychometric properties.[56] It measures physical functioning, role limitations, bodily pain, general health, vitality, social functioning, and mental health. SF-36v2 encompasses a physical component and a mental component summary.

The Depression Anxiety Stress Scale-21 (DASS-21)[57] comprises three 7-item subscales measuring depression, anxiety, and stress. Each item is scored on a 4-point Likert scale, with higher scores indicating more symptoms. The DASS-21 has good psychometric properties.[58]

The Multidimensional Fatigue Inventory (MFI-20)[59] will be used to measure fatigue. The questionnaire consists of 20 items that assess five dimensions of fatigue. A total score, ranging from 4 to 20, is calculated for each dimension by summation of the individual item scores. The Swedish version has good psychometric properties, for patients and healthy individuals.[60]

Clinical and sociodemographic data will be collected from ICs, including age, gender, marital status, relationship to the patient, children living at home, level of education, psychological support, employment, economic situation, and computer skills. Data about patients' age, gender, diagnosis and tumour stage, and treatment (surgery, radio therapy, and pharmacological treatment) will be collected from medical records.

During the individual interviews (described above), the ICs will be asked about their experiences of the questionnaires, e.g., 'What was your experience of answering the questionnaires?', 'Did you think the questions were relevant to answer initially and after a

For peer review only

For peer review only - http://bmjopen.bmj.com/site/about/guidelines.xhtml

month?', 'Did you find the questions easy to understand' and 'Do you have thoughts and feelings that we haven't asked you about?'.

Analysis

The quantitative data, e.g., demographic data and log data, will be analysed using descriptive statistics. The individual interviews will be analysed using qualitative content analysis following the approach of Graneheim and Lundman.[61] To confirm the rigorousness of the analysis the researchers will document and write extensive and detailed notes of the emergent analytical and theoretical insights.

Time plan

The preliminary timing of phase I is April 2021–March 2022, with phase II in March–November 2022.

The research team

Our research group was formed in 2020 and consists of HCPs from various fields and two experts in human-computer interaction. The members in the group have extensive experience of developing, testing, and evaluating internet-based complex interventions for ICs and patients.[62-65] Our group includes expertise in the field of human-computer interaction and user-centred design,[66] especially eHealth applications, including gender aspects,[67] expertise regarding ICs in the cancer context,[68] and expertise in nursing and medical aspects of patients with HNC.[69]

Patient and Public Involvement--

One of the researcher in the research team has own experience of being an IC to an individual treated for HNC. In addition we will form an expert panel with ICs to regularly advise the research group about the content and design of Carer eSupport and how the project should be executed. Further, we will invite an advisory board, consisting of researchers, managers in healthcare, politicians, and patient organization representatives to seminars, who can regularly provide their opinions on the project's progress and clinical relevance at seminars.

Discussion

Carer eSupport will be developed based on the experiences of ICs and HCPs, scientific evidence, SCT, and UTAUT. This will add to the theoretical basis regarding ICs' needs. The

For peer review only

goal is to develop a feasible and acceptable internet-based intervention to be evaluated in a forthcoming RCT.

We will perform a thorough review, as recommended by O'Cathain et al.[6] instead of following a systematic review methodology. Undertaking a systematic review is not always necessary when developing a complex intervention, since there may be recent reviews available – nor is it always possible, given limited resources. However, this may be a limitation, as we may overlook valuable information from past research.

A thorough conduction of phases I and II and establishment of an expert panel consisting of ICs and an advisory group, providing input on how the project can be carried out, will be crucial for the relevance of Carer eSupport. If the forthcoming RCT shows Carer eSupport to be effective regarding ICs' preparedness for caregiving and IC health, this will increase the probability that Carer eSupport can be implemented into healthcare.

Carer eSupport will be adapted to ICs who need to be prepared for caregiving at home due to the advanced treatment given to the individuals they provide care to.[11, 12] Engaging stakeholders, e.g., end users, in the development phase[6, 7] is therefore necessary to meet the ICs' needs. These stakeholders must have experience of caring for individuals with HNC at home, so they can find solutions that may make a difference for the ICs and identify and fill the gaps in relation to their needs. This is in line with the suggestions of Slev et al.[70]: that future studies should develop interventions and provide insight into the effects of eHealth on ICs in particular.

If a researcher does not perform a process evaluation when implementing a complex intervention, it is assumed that the implementation will proceed correctly, which rarely occurs. Therefore, we will perform a process evaluation. However, given the limited sample size, conclusions drawn should be considered with caution and supplemented with a process evaluation of the future RCT.

We are aware of the difficulties that may arise of conducting FGDs online. Some ICs may be unable to participate due to technical difficulties or not owning a computer. This may result in a lack of certain groups' experiences and needs. Also, patients with HNC make up a heterogenous group with differing needs and prognosis. We acknowledge this as a limitation. The intervention is adapted for ICs of individuals with HNC who have ongoing treatment or have been treated, and it might be a challenge to meet all their needs and expectations.

For peer review only - http://bmjopen.bmj.com/site/about/guidelines.xhtml

We believe that ICs of individuals with HNC are motivated to learn caregiving skills.[71] Previous research has shown that ICs experience lack of preparedness. Carer eSupport may provide ICs with support and opportunities to acquire new skills and increase their self-efficacy for caregiving. SCT[40] provides a useful framework for understanding the ICs' behaviours and needs.[41] According to SCT, self-efficacy and behavioural capabilities, such as perceived preparedness, as well as environmental factors, may be important predictors of IC distress and could be useful for identifying those who might benefit most from Carer eSupport. A strength is that we have complemented the theoretical framework with UTAUT,[42] to understand how each individual accepts technology. These theories serve as complementary tools for us to analyse the results and understand how to build Carer eSupport.

Ethics and dissemination

All procedures have been approved by the Swedish Ethical Review Authority at Uppsala University (Dnr: 2020-04650). Informed consent will be obtained before enrolment of patients, their ICs, and HCPs. Confidentiality will be ensured by replacing names and personal identity numbers with codes in all materials. The coding lists will be kept separately from other research materials. All study data will be treated as confidential information and stored in safe archives, available only to members of the research group. Results will be disseminated in peer-reviewed journal publications. The feasibility testing (phase II) is registered at Clinicaltrials.gov (Identifier: NCT05028452).

Funding statement

This work was supported by the Swedish Research Council (grant number 2019-01231), the Swedish Cancer Society (grant number 20 1014 PjF), the Oncology Department Foundations Research Fund in Uppsala (grant number N/a) and the Swedish state under the agreement between the Swedish government and the county councils, the ALF- agreement (Uppsala county: ALF-941900).

Competing interests statement

None declared.

Authors' contributions: BJ conceived the study, contextualised Carer eSupport, developed the protocol, is the grant holder of this study, and contributed critical input during drafting and finalization of the manuscript. All authors provided input on the design of Carer

For peer review only - http://bmjopen.bmj.com/site/about/guidelines.xhtml

eSupport. UL drafted and finalised the manuscript. YTE contributed with critical input during drafting and finalization of the manuscript. ÅC, MC, and AA provided critical input to finalise the protocol. GL and LvE contributed by critically commenting on and approving the final manuscript.

References

1. Craig, P., et al., *Developing and evaluating complex interventions: the new Medical Research Council guidance.* Bmj, 2008. **337**.
2. Hudson, P. and S. Payne, *The future of family caregiving: research, social policy and clinical practice.* Family Carers in Palliative Care: A guide for health and social care professionals, 2009: p. 2009.
3. Adashek, J.J. and I.M. Subbiah, *Caring for the caregiver: a systematic review characterising the experience of*

For peer review only

caregivers of older adults with advanced cancers. ESMO Open, 2020. **5**(5): p. e000862.

4. Craig, P., et al., *Developing and evaluating complex interventions: the new Medical Research Council guidance.* Bmj, 2008. **337**: p. a1655.
5. Skivington, K., et al., *A new framework for developing and evaluating complex interventions: update of Medical Research Council guidance.* bmj, 2021. **374**.
6. O'Cathain, A., et al., *Guidance on how to develop complex interventions to improve health and healthcare.* BMJ open, 2019. **9**(8): p. e029954.
7. Pandi-Perumal, S.R., et al., *Project Stakeholder Management in the Clinical Research Environment: How to Do it Right.* Frontiers in Psychiatry, 2015. **6**(71).
8. Crosby, R. and S.M. Noar, *What is a planning model? An introduction to PRECEDE-PROCEED.* Journal of public health dentistry, 2011. **71**: p. S7-S15.
9. DeLone, W.H. and E.R. McLean, *The DeLone and McLean model of information systems success: a ten-year update.* Journal of management information systems, 2003. **19**(4): p. 9-30.
10. Bray, F., et al., *Global cancer statistics 2018: GLOBOCAN estimates of incidence and mortality worldwide for 36 cancers in 185 countries.* CA: A Cancer Journal for Clinicians, 2018. **68**(6): p. 394-424.

For peer review only

11. Ehrsson, Y.T., P. Fransson, and S. Einarsson, *Mapping Health-Related Quality of Life, Anxiety, and Depression in Patients with Head and Neck Cancer Diagnosed with Malnutrition Defined by GLIM.* Nutrients, 2021. **13**(4): p. 1167.

12. Einarsson, S., G. Laurell, and Y. Tiblom Ehrsson, *Experiences and coping strategies related to food and eating up to two years after the termination of treatment in patients with head and neck cancer.* European journal of cancer care, 2019. **28**(2): p. e12964.

13. Wells, M., et al., *Distress, concerns and unmet needs in survivors of head and neck cancer: a cross-sectional survey.* European journal of cancer care, 2015. **24**(5): p. 748-760.

14. Ringash, J., et al., *Head and Neck Cancer Survivorship: Learning the Needs, Meeting the Needs.* Semin Radiat Oncol, 2018. **28**(1): p. 64-74.

15. Nund, R.L., et al., *Carers' experiences of dysphagia in people treated for head and neck cancer: a qualitative study.* Dysphagia, 2014. **29**(4): p. 450-8.

16. Patterson, J.M., et al., *Head and neck cancer and dysphagia; caring for carers.* Psychooncology, 2013. **22**(8): p. 1815-20.

17. Wang, T., S.R. Mazanec, and J.G. Voss. *Needs of Informal Caregivers of Patients With Head and Neck

For peer review only

Cancer: A Systematic Review. in *Oncology Nursing Forum*. 2021. Oncology Nursing Society.

18. Castellanos, E.H., et al., *Impact of patient symptoms and caregiving tasks on psychological distress in caregivers for head and neck cancer (HNC).* Psychooncology, 2019. **28**(3): p. 511-517.
19. Lee, C.Y., et al., *Depression, anxiety, quality of life, and predictors of depressive disorders in caregivers of patients with head and neck cancer: A six-month follow-up study.* J Psychosom Res, 2017. **100**: p. 29-34.
20. Schumacher, K.L., B.J. Stewart, and P.G. Archbold, *Conceptualization and measurement of doing family caregiving well.* Image J Nurs Sch, 1998. **30**(1): p. 63-9.
21. Given, B., et al., *Caregiver role strain.* Nursing Care of Older Adults diagnosis: Outcomes and Interventions. St. Louis, MO: Mosby, 2001: p. 679-95.
22. Goldstein, N.E., et al., *Factors associated with caregiver burden among caregivers of terminally ill patients with cancer.* J Palliat Care, 2004. **20**(1): p. 38-43.
23. Carter, P.A., *Caregivers' descriptions of sleep changes and depressive symptoms.* Oncol Nurs Forum, 2002. **29**(9): p. 1277-83.
24. Carrera, P.M., H.M. Kantarjian, and V.S. Blinder, *The financial burden and distress of patients with cancer: Understanding and stepping-up action on the financial*

For peer review only - http://bmjopen.bmj.com/site/about/guidelines.xhtml

toxicity of cancer treatment. CA Cancer J Clin, 2018. **68**(2): p. 153-165.

25. Ugalde, A., M. Krishnasamy, and P. Schofield, *Role recognition and changes to self-identity in family caregivers of people with advanced cancer: a qualitative study.* Support Care Cancer, 2012. **20**(6): p. 1175-81.
26. Mason, N. and S. Hodgkin, *Preparedness for caregiving: A phenomenological study of the experiences of rural Australian family palliative carers.* Health & social care in the community, 2019. **27**(4): p. 926-935.
27. Northouse, L.L., et al., *Interventions with family caregivers of cancer patients: meta-analysis of randomized trials.* CA Cancer J Clin, 2010. **60**(5): p. 317-39.
28. Applebaum, A.J. and W. Breitbart, *Care for the cancer caregiver: a systematic review.* Palliat Support Care, 2013. **11**(3): p. 231-52.
29. Ferrell, B. and E. Wittenberg, *A review of family caregiving intervention trials in oncology.* CA Cancer J Clin, 2017. **67**(4): p. 318-325.
30. Aung, S.H.H., K. White, and J. Bloomfield, *The Experiences and the Needs of Caregivers of Patients With Head and Neck Cancer: An Integrative Review.* Cancer Nursing, 2021. **44**(6): p. E361-E373.
31. D'souza, V., et al., *Multimedia information intervention and its benefits in partners of the head and neck cancer*

For peer review only - http://bmjopen.bmj.com/site/about/guidelines.xhtml

patients. European journal of cancer care, 2017. **26**(4): p. e12440.

32. Luo, X., et al., *A critical literature review of dyadic web-based interventions to support cancer patients and their caregivers, and directions for future research.* Psychooncology, 2020. **29**(1): p. 38-48.

33. Kaltenbaugh, D.J., et al., *Using Web-based interventions to support caregivers of patients with cancer: a systematic review.* Oncol Nurs Forum, 2015. **42**(2): p. 156-64.

34. Ahmad, A., P. Mozelius, and K. Ahlin. *Testbed Requirements for Technology Enhanced Stroke Rehabilitation to Support Independent Living*. in *ICT4AWE*. 2019.

35. Freund, M., et al., *Effectiveness of information and communications technology interventions for stroke survivors and their support people: a systematic review.* Disability and Rehabilitation, 2021: p. 1-16.

36. Faruque, M., et al. *Feasibility of Digital Health Services for Educating the Community People Regarding Lifestyle Modification Combating Noncommunicable Diseases*. in *International Conference on Human-Computer Interaction*. 2019. Springer.

37. Ugalde, A., et al., *A systematic review of cancer caregiver interventions: Appraising the potential for implementation*

For peer review only

of evidence into practice. Psycho-oncology, 2019. **28**(4): p. 687-701.

38. Firouzi, F., et al., *Ai-driven data monetization: The other face of data in iot-based smart and connected health.* IEEE Internet of Things Journal, 2020.
39. Cabestany, J., et al. *Artificial intelligence contribution to eHealth application*. in *2018 25th International Conference" Mixed Design of Integrated Circuits and System"(MIXDES)*. 2018. IEEE.
40. Schunk, D.H., *Social cognitive theory.* 2012.
41. Bandura, A., *Self-efficacy: toward a unifying theory of behavioral change.* Psychol Rev, 1977. **84**(2): p. 191-215.
42. Venkatesh, V., et al., *User acceptance of information technology: Toward a unified view.* MIS quarterly, 2003: p. 425-478.
43. Chan, A.-W., et al., *SPIRIT 2013 explanation and elaboration: guidance for protocols of clinical trials.* Bmj, 2013. **346**.
44. Creswell, J.W. and J.D. Creswell, *Research design: Qualitative, quantitative, and mixed methods approaches*. 2017: Sage publications.
45. Krueger, R.A. and M.A. Casey, *A practical guide for applied research.* A practical guide for applied research, 2000.

For peer review only - http://bmjopen.bmj.com/site/about/guidelines.xhtml

46. Lobe, B., D. Morgan, and K.A. Hoffman, *Qualitative Data Collection in an Era of Social Distancing.* International Journal of Qualitative Methods, 2020. **19**: p. 1609406920937875.

47. Braun, V. and V. Clarke, *Using thematic analysis in psychology.* Qualitative Research in Psychology, 2006. **3**(2): p. 77-101.

48. Lincoln, Y.S. and E.G. Guba, *But is it rigorous? Trustworthiness and authenticity in naturalistic evaluation.* New directions for program evaluation, 1986. **1986**(30): p. 73-84.

49. Sjöström, J., L. von Essen, and H. Grönqvist, *The origin and impact of ideals in eHealth research: experiences from the U-CARE research environment.* JMIR research protocols, 2014. **3**(2): p. e28.

50. The U-Care group. *Uppsala University Psychosocial Care Programme: U-CARE*. 2010 [cited 2016 Juni 15]; Available from: https://www.u-care.uu.se/u-care-portal.

51. Still, B. and K. Crane, *Fundamentals of user-centered design: A practical approach*. 2017: CRC press.

52. Cocks, K. and D.J. Torgerson, *Sample size calculations for pilot randomized trials: a confidence interval approach.* Journal of Clinical Epidemiology, 2013. **66**(2): p. 197-201.

53. Henriksson, A., et al., *Adaptation and psychometric evaluation of the Preparedness for Caregiving Scale,*

For peer review only

Caregiver Competence Scale and Rewards of Caregiving Scale in a sample of Swedish family members of patients with life-threatening illness. Palliat Med, 2012. **26**(7): p. 930-8.

54. Archbold, P.G., et al., *Mutuality and preparedness as predictors of caregiver role strain.* Research in nursing & health, 1990. **13**(6): p. 375-384.
55. Elmståhl, S., B. Malmberg, and L. Annerstedt, *Caregiver's burden of patients 3 years after stroke assessed by a novel caregiver burden scale.* Archives of physical medicine and rehabilitation, 1996. **77**(2): p. 177-182.
56. Taft, C., J. Karlsson, and M. Sullivan, *Performance of the Swedish SF-36 version 2.0.* Qual Life Res, 2004. **13**(1): p. 251-6.
57. Alfonsson, S., E. Wallin, and P. Maathz, *Factor structure and validity of the Depression, Anxiety and Stress Scale-21 in Swedish translation.* J Psychiatr Ment Health Nurs, 2017. **24**(2-3): p. 154-162.
58. Chiang, V.C.L., et al., *Fulfilling the psychological and information need of the family members of critically ill patients using interactive mobile technology: A randomised controlled trial.* Intensive Crit Care Nurs, 2017. **41**: p. 77-83.

For peer review only

59. Smets, E.M., et al., *The Multidimensional Fatigue Inventory (MFI) psychometric qualities of an instrument to assess fatigue.* J Psychosom Res, 1995. **39**(3): p. 315-25.

60. Hagelin, C.L., et al., *The psychometric properties of the Swedish Multidimensional Fatigue Inventory MFI-20 in four different populations.* Acta Oncol, 2007. **46**(1): p. 97-104.

61. Graneheim, U.H. and B. Lundman, *Qualitative content analysis in nursing research: concepts, procedures and measures to achieve trustworthiness.* Nurse education today, 2004. **24**(2): p. 105-112.

62. Cernvall, M., et al., *Twelve-Month Follow-Up of a Randomized Controlled Trial of Internet-Based Guided Self-Help for Parents of Children on Cancer Treatment.* J Med Internet Res, 2017. **19**(7): p. e273.

63. Wikman, A., et al., *Development of an Internet-Administered Cognitive Behavior Therapy Program (ENGAGE) for Parents of Children Previously Treated for Cancer: Participatory Action Research Approach.* J Med Internet Res, 2018. **20**(4): p. e133.

64. Woodford, J., et al., *Internet-administered cognitive behavioral therapy for common mental health difficulties in parents of children treated for cancer: intervention development and description study.* JMIR Formative Research, 2021. **5**(7): p. e22709.

For peer review only

65. Hauffman, A., et al., *Cocreated internet-based stepped care for individuals with cancer and concurrent symptoms of anxiety and depression: Results from the U-CARE AdultCan randomized controlled trial.* Psycho-Oncology, 2020. **29**(12): p. 2012-2018.

66. Gulliksen, J., et al., *Key principles for user-centred systems design.* Behaviour and Information Technology, 2003. **22**(6): p. 397-409.

67. Rexhepi, H., et al., *Cancer patients' attitudes and experiences of online access to their electronic medical records: A qualitative study.* Health informatics journal, 2018. **24**(2): p. 115-124.

68. Witkowski, Å. and M.E. Carlsson, *Support group programme for relatives of terminally ill cancer patients.* Supportive care in cancer, 2004. **12**(3): p. 168-175.

69. Stenhammar, C., et al., *Changes in intimate relationships following treatment for head and neck cancer-A qualitative study.* J Psychosoc Oncol, 2017. **35**(5): p. 614-630.

70. Slev, V.N., et al., *Effects of eHealth for patients and informal caregivers confronted with cancer: a meta-review.* International journal of medical informatics, 2016. **87**: p. 54-67.

71. Badr, H., et al., *Unmet needs and relationship challenges of head and neck cancer patients and their spouses.*

For peer review only

Journal of Psychosocial Oncology, 2016. **34**(4): p. 336-346.

For peer review only

For peer review only - http://bmjopen.bmj.com/site/about/guidelines.xhtml

Figure 1. Overview of the development phase and the feasibility testing phase of the intervention, Carer eSupport, according to Medical Research Councils guidelines for complex intervention.

For peer review only

For peer review only - http://bmjopen.bmj.com/site/about/guidelines.xhtml

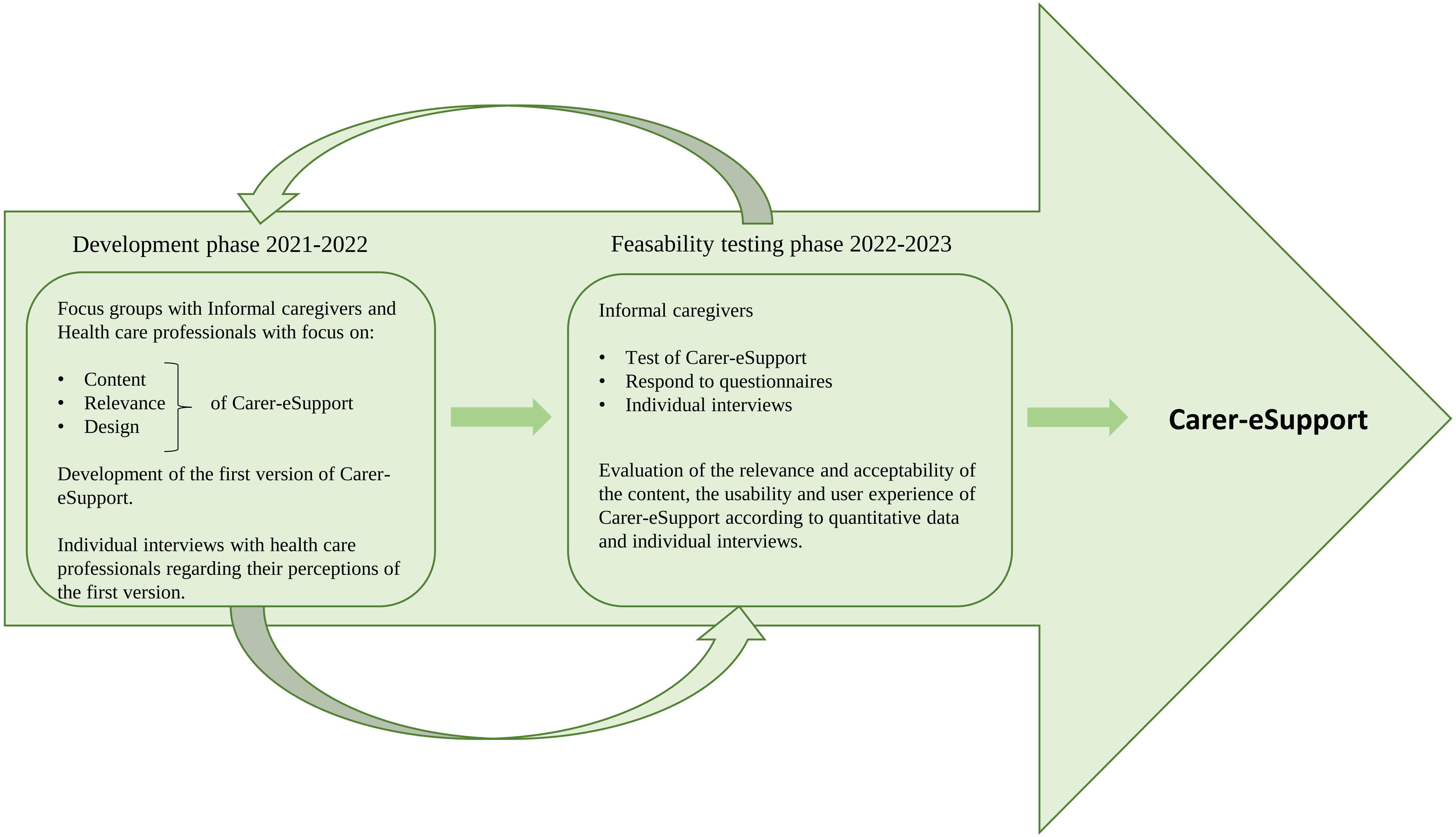
Development phase 2021-2022
Focus groups with Informal caregivers and Health care professionals with focus on:
• Content
• Relevance
• Design
of Carer-eSupport
Development of the first version of Carer-eSupport.
Individual interviews with health care professionals regarding their perceptions of the first version.
Feasability testing phase 2022-2023
Informal caregivers
• Test of Carer-eSupport
• Respond to questionnaires
• Individual interviews
Evaluation of the relevance and acceptability of the content, the usability and user experience of Carer-eSupport according to quantitative data and individual interviews.
Carer-eSupport

Reporting checklist for protocol of a clinical trial.

Based on the SPIRIT guidelines.

Instructions to authors

Complete this checklist by entering the page numbers from your manuscript where readers will find each of the items listed below.

Your article may not currently address all the items on the checklist. Please modify your text to include the missing information. If you are certain that an item does not apply, please write "n/a" and provide a short explanation.

Upload your completed checklist as an extra file when you submit to a journal.

In your methods section, say that you used the SPIRITreporting guidelines, and cite them as:

Chan A-W, Tetzlaff JM, Gøtzsche PC, Altman DG, Mann H, Berlin J, Dickersin K, Hróbjartsson A, Schulz KF, Parulekar WR, Krleža-Jerić K, Laupacis A, Moher D. SPIRIT 2013 Explanation and Elaboration: Guidance for protocols of clinical trials. BMJ. 2013;346:e7586

		Reporting Item	Page Number
**Administrative information**			
Title	#1	Descriptive title identifying the study design, population, interventions, and, if applicable, trial acronym	1

For peer review only

Trial registration	#2a	Trial identifier and registry name. If not yet registered, name of intended registry	1, 13<br>Clinicaltrials.gov. Feasibility study phase II.
Trial registration: data set	#2b	All items from the World Health Organization Trial Registration Data Set	N/a<br>The present study is feasibility study including informal caregivers and health professionals
Protocol version	#3	Date and version identifier	N/a. This is the only version.
Funding	#4	Sources and types of financial, material, and other support	13
Roles and responsibilities: contributorship	#5a	Names, affiliations, and roles of protocol contributors	Titel page, and p 14
Roles and responsibilities: sponsor contact information	#5b	Name and contact information for the trial sponsor	Titel page
Roles and responsibilities: sponsor and funder	#5c	Role of study sponsor and funders, if any, in study design; collection, management, analysis, and interpretation of data; writing of the report; and the decision to submit	N/a.

For peer review only

		the report for publication, including whether they will have ultimate authority over any of these activities	
Roles and responsibilities: committees	#5d	Composition, roles, and responsibilities of the coordinating centre, steering committee, endpoint adjudication committee, data management team, and other individuals or groups overseeing the trial, if applicable (see Item 21a for data monitoring committee)	N/a
**Introduction**			
Background and rationale	#6a	Description of research question and justification for undertaking the trial, including summary of relevant studies (published and unpublished) examining benefits and harms for each intervention	3
Background and rationale: choice of comparators	#6b	Explanation for choice of comparators	N/a. This is a feasibility study with an one arm feasibility study with a posttest design.
Objectives	#7	Specific objectives or hypotheses	5,6

For peer review only

Trial design	#8	Description of trial design including type of trial (eg, parallel group, crossover, factorial, single group), allocation ratio, and framework (eg, superiority, equivalence, non-inferiority, exploratory)	6
**Methods: Participants, interventions, and outcomes**			
Study setting	#9	Description of study settings (eg, community clinic, academic hospital) and list of countries where data will be collected. Reference to where list of study sites can be obtained	6
Eligibility criteria	#10	Inclusion and exclusion criteria for participants. If applicable, eligibility criteria for study centres and individuals who will perform the interventions (eg, surgeons, psychotherapists)	7,9
Interventions: description	#11a	Interventions for each group with sufficient detail to allow replication, including how and when they will be administered	8,9,10
Interventions: modifications	#11b	Criteria for discontinuing or modifying allocated interventions for a given trial	N/a

For peer review only

		participant (eg, drug dose change in response to harms, participant request, or improving / worsening disease)	
Interventions: adherance	#11c	Strategies to improve adherence to intervention protocols, and any procedures for monitoring adherence (eg, drug tablet return; laboratory tests)	Adherence will be assessed using log data from the portal and interviews.
Interventions: concomitant care	#11d	Relevant concomitant care and interventions that are permitted or prohibited during the trial	N/a
Outcomes	#12	Primary, secondary, and other outcomes, including the specific measurement variable (eg, systolic blood pressure), analysis metric (eg, change from baseline, final value, time to event), method of aggregation (eg, median, proportion), and time point for each outcome. Explanation of the clinical relevance of chosen efficacy and harm outcomes is strongly recommended	10
Participant timeline	#13	Time schedule of enrolment, interventions (including any run-ins and washouts), assessments, and visits for participants. A	Figure 1

For peer review only

		schematic diagram is highly recommended (see Figure)	
Sample size	#14	Estimated number of participants needed to achieve study objectives and how it was determined, including clinical and statistical assumptions supporting any sample size calculations	7
Recruitment	#15	Strategies for achieving adequate participant enrolment to reach target sample size	7, 9
**Methods: Assignment of interventions (for controlled trials)**			
Allocation: sequence generation	#16a	Method of generating the allocation sequence (eg, computer-generated random numbers), and list of any factors for stratification. To reduce predictability of a random sequence, details of any planned restriction (eg, blocking) should be provided in a separate document that is unavailable to those who enrol participants or assign interventions	N/a<br>This is a feasibility study with an uncontrolled feasibility study with a posttest design.

For peer review only

Allocation concealment mechanism	#16b	Mechanism of implementing the allocation sequence (eg, central telephone; sequentially numbered, opaque, sealed envelopes), describing any steps to conceal the sequence until interventions are assigned	N/a<br>This is a feasibility study with an uncontrolled feasibility study with a posttest design.
Allocation: implementation	#16c	Who will generate the allocation sequence, who will enrol participants, and who will assign participants to interventions	N/a
Blinding (masking)	#17a	Who will be blinded after assignment to interventions (eg, trial participants, care providers, outcome assessors, data analysts), and how	N/a
Blinding (masking): emergency unblinding	#17b	If blinded, circumstances under which unblinding is permissible, and procedure for revealing a participant's allocated intervention during the trial	N/a
**Methods: Data collection, management, and analysis**			
Data collection plan	#18a	Plans for assessment and collection of outcome, baseline, and other trial data,	10

For peer review only

		including any related processes to promote data quality (eg, duplicate measurements, training of assessors) and a description of study instruments (eg, questionnaires, laboratory tests) along with their reliability and validity, if known. Reference to where data collection forms can be found, if not in the protocol	
Data collection plan: retention	#18b	Plans to promote participant retention and complete follow-up, including list of any outcome data to be collected for participants who discontinue or deviate from intervention protocols	N/a<br>The aim of this feasibility study is to step by step thoroughly review all the processes and examine if intervention is relevant.
Data management	#19	Plans for data entry, coding, security, and storage, including any related processes to promote data quality (eg, double data entry; range checks for data values). Reference to where details of data management procedures can be found, if not in the protocol	8
Statistics: outcomes	#20a	Statistical methods for analysing primary and secondary outcomes. Reference to where other details of the statistical	11

For peer review only

		analysis plan can be found, if not in the protocol	
Statistics: additional analyses	#20b	Methods for any additional analyses (eg, subgroup and adjusted analyses)	8
Statistics: analysis population and missing data	#20c	Definition of analysis population relating to protocol non-adherence (eg, as randomised analysis), and any statistical methods to handle missing data (eg, multiple imputation)	N/a
**Methods: Monitoring**			
Data monitoring: formal committee	#21a	Composition of data monitoring committee (DMC); summary of its role and reporting structure; statement of whether it is independent from the sponsor and competing interests; and reference to where further details about its charter can be found, if not in the protocol. Alternatively, an explanation of why a DMC is not needed	N/a
Data monitoring: interim analysis	#21b	Description of any interim analyses and stopping guidelines, including who will have access to these interim results and	N/a

For peer review only

		make the final decision to terminate the trial	
Harms	#22	Plans for collecting, assessing, reporting, and managing solicited and spontaneously reported adverse events and other unintended effects of trial interventions or trial conduct	N/a
Auditing	#23	Frequency and procedures for auditing trial conduct, if any, and whether the process will be independent from investigators and the sponsor	N/a
**Ethics and dissemination**			
Research ethics approval	#24	Plans for seeking research ethics committee / institutional review board (REC / IRB) approval	13
Protocol amendments	#25	Plans for communicating important protocol modifications (eg, changes to eligibility criteria, outcomes, analyses) to relevant parties (eg, investigators, REC / IRBs, trial participants, trial registries, journals, regulators)	13
Consent or assent	#26a	Who will obtain informed consent or assent from potential trial participants or	6,13

For peer review only

		authorised surrogates, and how (see Item 32)	
Consent or assent: ancillary studies	#26b	Additional consent provisions for collection and use of participant data and biological specimens in ancillary studies, if applicable	N/a
Confidentiality	#27	How personal information about potential and enrolled participants will be collected, shared, and maintained in order to protect confidentiality before, during, and after the trial	13
Declaration of interests	#28	Financial and other competing interests for principal investigators for the overall trial and each study site	13
Data access	#29	Statement of who will have access to the final trial dataset, and disclosure of contractual agreements that limit such access for investigators	13,14<br>N/a. Data will only be handled in the research group.
Ancillary and post trial care	#30	Provisions, if any, for ancillary and post-trial care, and for compensation to those who suffer harm from trial participation	N/a
Dissemination policy: trial results	#31a	Plans for investigators and sponsor to communicate trial results to participants,	13

For peer review only

		healthcare professionals, the public, and other relevant groups (eg, via publication, reporting in results databases, or other data sharing arrangements), including any publication restrictions	
Dissemination policy: authorship	#31b	Authorship eligibility guidelines and any intended use of professional writers	14
Dissemination policy: reproducible research	#31c	Plans, if any, for granting public access to the full protocol, participant-level dataset, and statistical code	Present study protocol will be published with open access.

Appendices

Informed consent materials	#32	Model consent form and other related documentation given to participants and authorised surrogates	6<br>N/a<br>The participants confirm participation with a study specific informed consent.
Biological specimens	#33	Plans for collection, laboratory evaluation, and storage of biological specimens for genetic or molecular analysis in the current trial and for future use in ancillary studies, if applicable	N/a

For peer review only

None The SPIRIT Explanation and Elaboration paper is distributed under the terms of the Creative Commons Attribution License CC-BY-NC. This checklist can be completed online using https://www.goodreports.org/, a tool made by the EQUATOR Network in collaboration with Penelope.ai

For peer review only
